# Anthropogenic hybridization and its influence on the adaptive potential of the Sardinian wild boar (*Sus scrofa meridionalis*)

**DOI:** 10.1007/s13353-023-00763-x

**Published:** 2023-06-28

**Authors:** Giulia Fabbri, Ludovica Molinaro, Nadia Mucci, Luca Pagani, Massimo Scandura

**Affiliations:** 1 Department of Veterinary Medicine, University of Sassari, Via Vienna 2A, 07100 Sassari, Italy; 2 Department of Human Genetics, KU Leuven, 3000 Leuven, Belgium; 3 Estonian Biocentre, Institute of Genomics, University of Tartu, Riia 23b, 51010 Tartu, Estonia; 4 Unit for Conservation Genetics (BIO-CGE), Italian Institute for Environmental Protection and Research (ISPRA), Ozzano dell’Emilia, Bologna, Italy; 5 Department of Biology, University of Padua, Viale G. Colombo 3, 35131 Padua, Italy

**Keywords:** *Sus scrofa*, SNPs, Introgression, Local ancestry inference, Selection

## Abstract

The wild boar (*Sus scrofa meridionalis*) arrived in Sardinia with the first human settlers in the early Neolithic with the potential to hybridize with the domestic pig (*S. s. domesticus*) throughout its evolution on the island. In this paper, we investigated the possible microevolutionary effects of such introgressive hybridization on the present wild boar population, comparing Sardinian wild specimens with several commercial pig breeds and Sardinian local pigs, along with a putatively unadmixed wild boar population from Central Italy, all genotyped with a medium density SNP chip. We first aimed at identifying hybrids in the population using different approaches, then examined genomic regions enriched for domestic alleles in the hybrid group, and finally we applied two methods to find regions under positive selection to possibly highlight instances of domestic adaptive introgression into a wild population. We found three hybrids within the Sardinian sample (3.1% out of the whole dataset). We reported 11 significant windows under positive selection with a method that looks for overly differentiated loci in the target population, compared with other two populations. We also identified 82 genomic regions with signs of selection in the domestic pig but not in the wild boar, two of which overlapped with genomic regions enriched for domestic alleles in the hybrid pool. Genes in these regions can be linked with reproductive success. Given our results, domestic introgression does not seem to be pervasive in the Sardinian wild boar. Nevertheless, we suggest monitoring the possible spread of advantageous domestic alleles in the coming years.

**Supplementary Information:**

The online version contains supplementary material available at 10.1007/s13353-023-00763-x.

## Introduction

Domestication is the process by which a limited number of individuals from a wild population are selected to obtain desirable changes in behavior, morphology, and physiology (Darwin 1868; Andersson and Georges 2004; Ramos-Onsins et al. 2014; Frantz et al. 2015; Ostrander et al. 2017). The loci governing the variability at the selected phenotypes will show a selective sweep in the domestic population after some time. If hybridization between the wild and domestic forms occurs — a case of anthropogenic hybridization — the hybrids can generally backcross into the wild population, as the divergence between the two populations is low enough to produce fertile offspring. The consequences of possible introgression of domestic alleles into the wild populations are difficult to predict: on the one hand, anthropogenic hybridization is considered a threat for the conservation of wild species because of the possible genetic swamping in wild populations (Todesco et al. 2016; McFarlane and Pemberton 2019), on the other hand, there could be domestic alleles that are advantageous in the wild. Examples of adaptive introgression were discovered, for instance, in the wolf (Anderson et al. 2009; Pilot et al. 2021): it was recently demonstrated that a dog-derived 3-bp deletion within the β-defensin gene CBD103 conferred resistance to the canine distemper virus in those areas interested by frequent outbreaks in North America (Cubaynes et al. 2022).

The wild boar (WB; *Sus scrofa*, Linnaeus 1758) was likely domesticated at multiple centers in Eurasia around 10,000 years ago (Larson et al. 2010; Ottoni et al. 2013; Frantz et al. 2019; Zeder and Lemoine 2022). Gene flow between the WB and the domestic pig (DP, *S. scrofa domesticus*) has seemingly occurred for a long time (Frantz et al. 2015) and was drastically reduced only from the XVII–XIX centuries on, when a substantial shift in pig husbandry in Europe led to the formation of the modern pig breeds.

The WB has been thriving on the island of Sardinia since the early Neolithic, most likely arriving with the first Neolithic settlers (Albarella et al. 2006). It was suggested that the first boars on the islands were primordial forms of DP and that they subsequently escaped and became feral. The Sardinian WB (SarWB) is classified as a different subspecies (*S. s. meridionalis*) from populations distributed throughout Europe (*S. s. scrofa*). Its evolution on the island is characterized by the coexistence with the DP, in particular with the local stocks that have been kept in semi-open or open conditions until such husbandry practices were banned in 2012 (Bozzi et al. 2019). This condition clearly increased the chances of hybridization between the wild and domestic forms in the natural environment. Moreover, intentional crosses are occasionally conducted within farms, but this source of hybridization does not seem to be prevalent in Sardinia (Canu et al. 2014). A previous work on a small sample of Sardinian WB analyzed the level of hybridization at several thousand of single nucleotide polymorphisms (SNPs) and classified 12% of the individuals as potential hybrids (Iacolina et al. 2018).

The role of adaptive introgression in the demographic success of the WB is still understudied. Fulgione et al. (2016) pointed out an interesting relationship between the hybrid status and the litter size of 62 WBs in Southern Italy, with the hybrids having more piglets. It is possible that the demographic recovery of the WB during the last century in Sardinia as elsewhere in Europe (Massei et al. 2015) was influenced by the presence of advantageous alleles of domestic origin in the population.

Here, we increased the sample size with respect to Iacolina et al. (2018) to better characterize the level of hybridization in the WB on the island of Sardinia; we applied an admixture deconvolution approach to describe the genetic landscape of domestic introgression; finally, we looked for signature of selection in the regions that we found enriched for DP alleles. Our focus was directed to recent hybridization events rather than to the whole history of hybridization on the island of Sardinia, as the prolonged time of possibly repeated admixture events at an early stage of WB and DP coexistence on the island makes it hard to identify events that occurred far in the past. Our findings enrich our knowledge on the effects of anthropogenic hybridization on wildlife populations. Moreover, the discovery of putative introgressed domestic alleles with a positive effect on fitness should encourage the monitoring of their spread in the population with follow-up campaigns to be included in the management of WB in Sardinia.

## Methods

### Dataset collection and quality control

The dataset we analyzed here was taken from publicly available data (Iacolina et al. 2016; Yang et al. 2017; Scandura et al. 2022), comprising various WB, DP, and outgroup species, all genotyped with the Illumina Porcine SNP60 Beadchip (Ramos et al. 2009), that is mapped onto the pig reference genome Sscrofa10.2 (Groenen et al. 2012). In particular, we selected the SarWB samples, together with a reference set of commercial European pig breeds (Berkshire (BK), Duroc (DU), Large White (LW), Pietrain (PI), Yorkshire (YO), Sardinian local pigs (SarDP)), and Italian WB that are assumed to be free of recent domestic introgression (ItaWB), and finally an outgroup — here represented by *S. barbatus* (SB; Bornean bearded pig). The merged dataset (Table 1) underwent a quality control (QC) step in PLINK 1.9 (Purcell et al. 2007) to remove the sites with at least 10% missing genotype rate, with low allele frequency (MAF < 0.05), and in linkage disequilibrium (LD, *r*^2^ < 0.5). We retained 38K SNPs to use in the structure analyses.

**Table 1:** The merged dataset gathered for this study

Population	Type	Sample size
SarWB	Wild boar	96
ItaWB	Wild boar	19
BK	Domestic pig	10
DU	Domestic pig	10
LW	Domestic pig	10
PI	Domestic pig	10
YO	Domestic pig	10
SarDP	Domestic pig	8
SB	Outgroup	11

### Admixture analysis

The identification of possible hybrids in the SarWB sample was achieved using different methods. We first ran a principal component analysis (PCA) with *smartpca* software available in EIGENSOFT v7.2.0 (Price et al. 2006; Patterson et al. 2006). We used all samples except for SarWB to define the PC space, and then projected the target WB in the defined space using the option “lsqproject.” The reasons behind this choice are: (i) avoiding that the number of Sardinian samples (higher than the other samples) biased the PC space; (ii) avoiding that the genetic patterns of the Sardinian samples biased the PC space, and thus directly testing how their genetic variance relates to the other samples. Then, we applied the model-based method implemented in the software ADMIXTURE v1.3 (Alexander et al. 2009) to assign individual ancestry coefficients, according to a specified number of ancestral populations. We tested *K* = 2−15 and computed the cross-validation error for each *K* value. After inspecting the results, we split the putative hybrids from their own group, thus creating the sub-groups Hybrid_SarWB within the Sardinian WB (i.e., the target population). Other two populations showed samples with a distinctive (possibly admixed) identity: the Sardinian local pigs (SarDP) and the outgroup Bornean bearded pig (SB); so, we defined Outlier_SarDP and Outlier_SB respectively.

We computed all the pairwise *F*_ST_ among the WB populations and DP breeds in our dataset with EIGENSOFT with the *smartpca* command using the “fstonly:YES” option. Hybrid_SarWB samples were analyzed separately from the putative pure SarWB.

### Introgression analysis

We formally tested the introgression of domestic alleles into the SarWB sample with f3-admixture statistics using AdmixTools v650 software with “qp3Pop” command (Patterson et al. 2012). Given two reference populations, the test infers whether the target population has arisen from the admixture of the two references. An admixture event is indicated by negative f3 results and Z-scores < −3. We performed the analyses using a dataset that has not gone through QC in order to retain the maximum number of SNPs (i.e., 54 K); LD is accounted for in the test with a jackknife procedure. We first considered the whole SarWB as the target and tested, if it could be the result of the pure WB population from ItaWB, and all the DP breeds tested individually. Then, we modeled the Hybrid_SarWB subgroup as admixture between the putative pure SarWB and each DP breed. We checked that none of the Sardinian samples included as source population showed signs of introgression with the DP by using a leave-one-out approach as follows: f3(DP, SarWB_all-i_, SarWB_i_). Thus the second source, SarWB_all-i_, is made of all putatively pure SarWB individuals except the one tested as target.

We examined as well the nature of SarDP to better clarify its origin and possibly ascertain a wild genetic introgression in Outlier_SarDP.

We explored with “qpGraph” v735 (Patterson et al. 2012) the relations among the populations included in the study, focusing on the positioning of SarWB with respect to the domestic cluster because of the common hypothesis about the origin of the population that involves the feralization of a primordial domestic form arrived from the mainland with the Neolithic settlers (Albarella et al. 2006).

### Local ancestry analysis

Dating the admixture event improves the local ancestry (LA) analyses with ELAI (Guan 2014). Therefore, we took advantage of the software MALDER (Loh et al. 2013), which is able to detect multiple admixture events based on the LD decay. The highest values of amplitude indicated the most suited reference sources for the admixture event given the LD decay.

Furthermore, to assign wild or domestic ancestry to genome stretches of the introgressed samples, we used the LA inference algorithm implemented in ELAI. We carried out the phasing step in SHAPEIT v2, following the recombination map of Tortereau et al. (2012), and set the admixture generations parameter to 20. We set the Hybrid_SarWB samples as the admixed group along with other two non-admixed SarWB samples as a control. As many other tools, ELAI reaches higher performances when several individuals are included in the analyses. Therefore, we clustered three groups of pigs rather than only supply one group as source (*N* < 10). In particular, LW and YO were indicated as proxy sources for the pig ancestry in f3-admixture analysis (Online Resource 1: Table S1). Additionally, we included as third breed PI rather than SarDP because even though *F*_ST_ is lower between SarDP and the proxy sources, the breed is a deeply heterogeneous group (see ADMIXTURE results in “Admixture analysis”), so PI was the second best option among all pairwise *F*_ST_ comparisons with LW and YO. The program returns the LA assignment for both the paternal and the maternal haplotypes. We ran ELAI on each chromosome 10 times and then averaged the results of the runs; each of the 10 runs went through an EM algorithm 20 times. We applied an assignment threshold of 0.9, all sites that could not reach the threshold were labeled as “unassigned” and were excluded for a given individual from the downstream analyses that rely on LA assignment.

Since we were interested in identifying regions across the genome of the introgressed individuals that can be ascribed to the domestic population, we then identified the windows where the proportion of domestic assignment was consistently high in the sampled hybrids (i.e., at least two-thirds of the haplotypes we analyzed were assigned to the domestic pool).

### Selection scan and gene ontology

As we wanted to describe possible patterns of adaptive introgression from the DP into the Sardinian WB population, we applied two different methods to identify signals of positive selection: Population Branch Statistics (PBS) and cross-population extended haplotype homozygosity (XP-EHH). PBS (Voight et al. 2006) is based on three populations and the underlying idea of the method is that sites where the target population shows higher distance (calculated from pairwise *F*_ST_ indexes) from the shared common source with the other two should be under divergent selection. We compared Hybrid_SarWB with putatively pure SarWB and ItaWB representing the wild components. We first computed pairwise *F*_ST_ per site with VCFTools v0.1.17 (Danecek et al. 2011), then we transformed the index into pairwise distances with the following formula:

$$T\left(i,j\right)=-\log \left(1- Fst\left(i,j\right)\right)$$

where *i* and *j* are all the possible pairs between Hybrid_SarWB, SarWB, and ItaWB. Next, we calculated the distance of Hybrid_SarWB from the other two WB populations as follows:

$$PBS=\frac{T(AB)+T(AC)-T(BC)}{2}$$

where *A* is the target population Hybrid_SarWB; *B* is represented by the pure SarWB; and *C* is ItaWB. We considered as candidate loci those above the 95th percentile of the genome-wide PBS distribution and then focused only on the windows with at least 5 consecutive SNPs above the threshold.

Furthermore, we wanted to check whether the results we obtained could be just the effect of the reduced sample size of the introgressed WB. Hence, we implemented two sets of randomizations (see Online Resource 2: Suppl. Text for details).

Considering the difficulty in detecting signatures of selection in admixed populations (e.g., Yelmen et al. 2021), we decided to look at the genomic signatures of positive selection in the putative domestic source of introgression and then check for enrichment of domestic alleles in those regions. Hence, we applied XP-EHH analysis (Sabeti et al. 2007) to the pool of domestic breeds used for LA (i.e., LW, PI, and YO) and compared it with the pure SarWB sample. XP-EHH relies on the pattern created by linkage between the variant under selection and its surroundings: a genomic region is expected to show longer haplotypes in the population where the alleles are advantageous. This method is ideal to detect recent selection as recombination breaks down the haplotype length after several generations. We used *rehh* R package v3.2.2 (Gautier et al. 2017) to apply this statistic on the phased dataset of DP and unadmixed SarWB (Online Resource 2: Suppl. Text).

We focused on the windows that intersected between one of the selection analyses and the ELAI results. We looked for transcripts falling within 100 kbp around the marker with the highest XP-EHH value, to account for linkage disequilibrium, using the NCBI RefSeq table on the UCSC website (https://genome.ucsc.edu/cgi-bin/hgTables accessed on 23 June 2022) (Karolchik et al. 2004) with the annotation available for Sscrofa10.2 genome build. We also looked at the genes falling within the significant windows identified only with ELAI or PBS.

## Results

### Population structure and differentiation

**Fig. 1:**
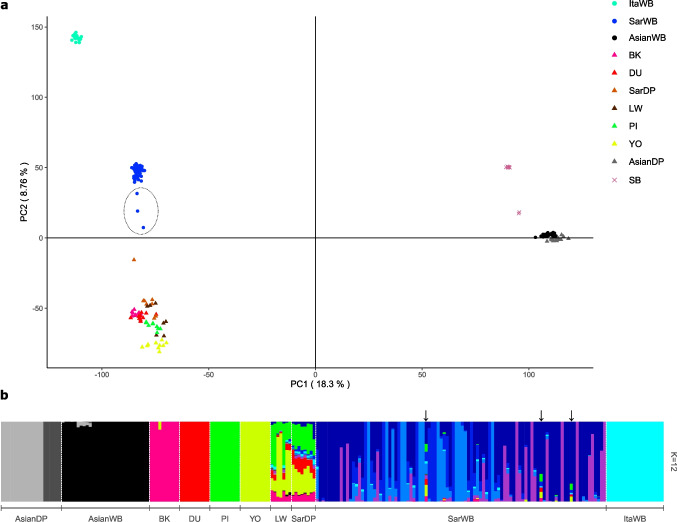
Genetic structure of the full dataset considered in the study. **a** Principal component analysis (PCA) and **b** admixture analysis on the Sardinian wild boar (SarWB), compared with Italian and Asian wild boars (ItaWB and AsianWB) and a set of commercial and local pig breeds. The number of ancestral populations is *K* = 12. The three hybrid SarWB are circled in the PCA and identified by arrows in the ADMIXTURE barplot. SarWB, Sardinian wild boar (WB); ItaWB, Italian WB; AsianWB, Asian WB; BK, Berkshire; DU, Duroc; LW, Large White; PI, Pietrain; YO, Yorkshire; SarDP, Sardinian local pig; AsianDP, Meishan pig breed from Asia

The PCA space described by the samples was consistent with Yang et al. (2017) results (Fig. 1a). Notably, three Sardinian samples were drawn towards the DP cluster, indicating a level of substructure within the Sardinian boar population; we classified them as Hybrid_SardWB and targeted them in the following analyses. Additionally, one SarDP individual, separated from its cluster, fell between the SarDP group and SarWB cluster. Two SB individuals did not fall within their cluster.

From the admixture analysis (Online Resource 2: Fig. S1), *K* = 12 (Fig. 1b) was the most supported number of ancestral components according to the cross-validation error (Online Resource 2: Fig. S2). At this point all the major populations/breeds were separated, with SarDP and LW showing a mosaic of contributions and SarWB showing internal substructure. Interestingly, the *F*_ST_ between the Sardinian WB and the three introgressed WB from the same population was pretty high (*F*_ST_ = 0.08), a signal of population divergence (Hartl et al. 1997).

### Hybrid detection and local ancestry inference

We performed f3-admixture analyses aiming to model the Sardinian boars as a mixture of a boar-like ancestry and a pig-like ancestry. We first analyzed the samples population-wide (Online Resource 1: Table S2) and then the three Hybrid_SarWB samples that emerged in the previous analyses (Online Resource 1: Table S1). No admixture was found when we tested SarWB as a mixture of ItaWB and pig-like ancestry. On the other hand, we found at least four putative triads that yielded significant results when analyzing together the three outlier Sardinian WB: (Hybrid_SarWB;YO,SarWB) and (Hybrid_SarWB;LW,SarWB), (Hybrid_SarWB;AsianWB,SarWB), and (Hybrid_SarWB;AsianDP,SarWB), with SarWB being all the putatively pure Sardinian WB samples. When using a leave-one-out approach to check for traces of domestic introgression in every single individual in the Sardinian wild boar group that we used as pure reference we did not highlight any significant result (Online Resource 1: Table S3). An intriguing result about the Sardinian WB population was obtained when we modeled the relationships among the populations included in the study, as we found that a tree placing SarWB branch as originating from the shared node from which all the DP breeds then departed with a high genetic drift (Online Resource 2: Fig. S4) was not rejected by “qpGraph.”

Additionally, given the low *F*_ST_ value between SarDP and LW and the high level of crossbreeding that characterized the history of the Sardinian breed in the last century (Albarella et al. 2007), we tried to model SarDP as a mixture of different pig sources. We found a significant result when modeling SarDP as admixture between DU and LW (Online Resource 1: Table S2). With regard to the Outlier_SarDP individual instead, although we hypothesized that its distinctiveness is derived from an admixture event with the Sardinian WB, the f3-admixture analysis did not return any significant result (Online Resource 1: Table S4).

Using MALDER, we retrieved one admixture event in the Hybrid_SawWB sample, dated around ~20 generations ago (Online Resource 1: Table S5). Applying a generation time of 5 years (Groenen 2016), this means that we are looking at an admixture event dated back 100 years ago. We can assume that MALDER was not able to detect multiple admixture events due to the small number of SNPs we were analyzing.

Global proportions of domestic contribution estimated from LA revealed non-uniform levels of introgression in the available set of Sardinian WB hybrids, ranging from 10 to 26.6% (Online Resource 1: Table S6). Nonetheless, chromosomes 4, 7, 13, and 14 were the most affected, while chromosome 18 the least (Online Resource 3: Fig. S5; the caption of this Fig. is in Online Resource 2). We did not find any window with 100% domestic assignment, so we report windows with domestic ancestry proportions: (1) greater than 67% (i.e., 4 out of 6 chromosomes evaluated, hereafter referred as p0.67; Table 2) and (2) greater than 83% (i.e., 5 out of 6 chromosomes evaluated, hereafter referred as p0.83; Online Resource 1: Table S7). The longest windows were found on chromosomes 13 and 14 (around 20 Mbp long), followed by chromosome 4 (17 Mb), 1 (12 Mb), and 6 (12 Mb).

**Table 2:** Genomic windows with high domestic proportion. Windows with domestic ancestry in 67% of the chromosomes analyzed (p0.67). In bold, the windows that intersected with at least one of the selection analyses (see below)

Chromosome	Start (bp)	Stop (bp)
1	12682937	24505411
4	21965834	22030619
4	60613361	77293029
5	9320895	17310489
6	126553874	138479475
6	142452858	143263519
6	148290171	152208520
7	3273939	7698093
7	79711030	81660424
7	124385985	127564283
10	73397126	73445140
**13**	**84062043**	**105334978**
**14**	**21902332**	**41317883**
14	75445007	78101592

### Signatures of selection

PBS identified 11 genomic regions differentiated in Hybrid_SarWB with respect to the two wild populations SarWB and ItaWB (Online Resource 1: Table S8). However, none of those were in common with the domestic regions found by ELAI. Chromosome 7 had the highest number of outlier windows, each of some hundreds kbp, followed by chromosome 14. The average number of significant windows found with PBS on the two randomization sets was 7.99 and 7.74, respectively. Four windows repeatedly resulted significant in the randomization replicates, one on chromosome 6, one on chromosome 7, and two on chromosome 14; so we consider them as not reliable (Online Resource 1: Table S8).

XP-EHH found 82 genomic windows under positive selection in the DP and not in the pure SarWB sample (Online Resource 1: Table S9). Chromosome 14 showed the highest number of windows (11), followed by chromosomes 1 and 8 (9 windows each). Two windows intersected regions identified as enriched for domestic alleles in the p0.67 list: one on chromosome 13 and one on chromosome 14 (Online Resource 1: Table S10). Interestingly, one region on chromosome 7 (32.5–33.0 Mbp) was in common with PBS but did not show an excess of domestic alleles in the introgressed group. By inspecting the haplotype structure around the region, we found that of 6 haplotypes from the Hybrid_SarWB sample, 4 belonged to the most common haplotypes in the Sardinian WB, while the other two are very similar to the haplotypes found in various DP breeds (Online Resource 2: Fig. S6, the caption of this Fig. is in Online Resource 2), consistent with the LA assignation. When we zoomed out of the region, extending for 2 Mb on each side, we noticed that the region was characterized by a reduced diversity, compared to the surrounding regions (Online Resource 2: Fig. S7, the caption of this Fig. is in Online Resource 2).

The annotation on Sscrofa10.2 reference genome for the two regions on chromosomes 13 and 14 identified two predicted genes on chromosome 13: P2RY214, a G-protein coupled purinergic receptor, and MED12L, which produces a coactivator implied in the transcriptional activation of many RNA polymerase II-dependent genes. The gene lists obtained from the separate annotation of the windows significant only in ELAI or PBS analyses resulted, as enriched in many metabolic GO terms in both cases, but there was no overlap in molecular functions or biological processes (Online Resource 1: Tables S11 and S12).

## Discussion

In the present study, we investigated the pattern of DP introgression into the Sardinian WB. We found that the percentage of hybrids was around 3.1%, which is less than what was reported in a previous work on a reduced dataset (Iacolina et al. 2018). Other studies focused on specific regions in Europe: Goedbloed et al. (2013) found that around 10% of the wild boar included in their study (*N* = 88) carried domestic alleles in an area encompassing the Netherlands, Luxemburg, and Western Germany, while Mary et al. (2022) reported a percentage of recent hybrids comparable to ours (3.6%) in a large French sample (*N* = 362). We were interested in detecting relatively recent hybrids because of the prolonged coexistence of Sardinian WB and DP on the island and the traditional pig husbandry that could have facilitated several historical admixture events, followed by backcrosses in the wild population. The three hybrid WB that we identified showed clear signs of domestic introgression in all the analyses we applied.

With regard to the use of the rest of the Sardinian WB sample as “pure” reference population in the LA analysis, we think that it was the most appropriate choice because: (I) we tested one by one the pure SarWB samples with the f3 statistics and did not find any significant result; (II) we included a subset of SarWB in the target group of the LA analysis and got negligible proportion of genome-wide domestic assignment. It is noteworthy that when we modeled the relationships among our wild and domestic populations in “qpGraph,” we observed that the Sardinian WB could well stem from a basal node, here labeled the “Domestication” node. As it is generally reported that the origin of this insular population could come from primordial domesticated form arrived with the Neolithic people and feralized after escaping (Albarella et al. 2006), it is tempting yet in need of further analysis to see a parallelism between this pattern, that may point to the Sardinian wild boar population as a relic of early Neolithic DPs, and the genome of human populations inhabiting the island, which shows a remarkable continuity with the one of the first Neolithic populations that arrived from West Asia (Mathieson et al. 2015). The admixture graph analysis here presented was intended to solely test whether the data could support a historical close relationship between SarWB and DP in general; this relationship with the DP deserves further investigation with the help of whole genome sequencing data and possibly ancient data, but for the sake of our present work we wanted to focus on the introgressive hybridization that involved modern improved DP breeds, as obtained from the XIX century onwards (Bosse et al. 2014). Another remark in this regard concerns the traditional semi-open husbandry of the DP that has been practiced in Sardinia for a long time and until the last decade: if after historical and repeated admixture between Sardinian WB and DP some genomic regions of domestic origin introgressed and spread in the population, it is possible that we missed a few windows of ancient domestic origin as the capacity of discrimination would be extremely low. Schleimer et al. (2022) used a pool of central European populations of WB as reference for the boar-like ancestry in the LA inference in a sample of Corsican and Sardinian WB. However, using a mix of populations as a reference for this analysis is not recommended, because the program ELAI is based on the pattern of LD decay, which is population-specific and is influenced by many factors including demographic history (Wright et al. 2005). Moreover, we cannot be sure of the purity of other WB populations; in fact WB from central Europe are affected by domestic introgression (Frantz et al. 2013; Goedbloed et al. 2013; Iacolina et al. 2018). The other reference pool in our LA analysis, representing the domestic component, did not include the Sardinian pigs: considering the admixed ancestry profile (Fig. 1b and Online Resource 1: Table S2) we preferred to use commercial breeds instead.

It is interesting that the Asian WB and DP used to model hybridization in the Sardinian WB were equally good in explaining the genetic composition of the Hybrid_SawWB. Mary et al. (2022) found that the major domestic component in the hybrids they identified had Asian origin. This can be due to the occurrence of Asian genomic material in the gene pool of several European DP breeds (Bosse et al. 2014). However, the ascertainment bias introduced by the commercial SNPchip used in this study seems to limit our ability to attribute the Asian component to either the wild or the domestic Asian lineages.

The percentage of genome-wide domestic assignment varied in the three samples but was quite high (above 10% in all cases). Notably, the expected domestic proportion after only three generations of backcrosses into the wild population should be around 12.5% and it halves after every generation; we found that the admixture event was much older, so it seems that some portions of domestic origin are being retained. We should, however, treat with caution the admixture time obtained from MALDER, as the medium-density SNP dataset analyzed here does not guarantee precise estimates. When we looked in particular at the regions where the domestic ancestry was high in the overall introgressed sample (p0.67 and p0.83), we found some chromosomes standing out for their pattern. It is noteworthy that chromosome 13 had lower WB ancestry in Mary et al. (2022) too, but not in Schleimer et al. (2022); perhaps the use of the direct population of origin of the introgressed group rather than a proxy allowed to detect different signals.

We identified overly differentiated windows in Hybrid_SarWB, compared to the two wild populations used as reference (i.e., pure SarWB and ItaWB) with PBS analysis. This statistic is based on *F*_ST_ between populations, whose estimate requires a minimum number of individuals in each population to be accurate — as low as 4–6, according to Willing et al. (2012). In our study case, there were only three individuals representing the introgressed group: as the allele frequencies derived from such a reduced number can be biased, we considered only the regions that were not found by the randomizations.

The other approach we used to look for selection, XP-EHH, was applied to compare the source populations in order to overcome the little introgressed representation. The underlying assumption with this approach is that some of the genes under selection in the DP give an immediate advantage to the carriers in the WB population, i.e., the coefficient of selection is very high. Therefore, once an advantageous allele enters the population, it spreads rapidly. If recombination around the locus had the chance to break the initially longer introgressed haplotype, what can happen is that XP-EHH identifies the locus as significant because the DP population is subjected to both natural and artificial selection while the WB only to the former one, but PBS will not show a long branch in correspondence of the locus because the domestic alleles have entered into the “wild” population too. This may explain the little overlap between the two methods.

The two genes that we recovered close to the significant markers from XP-EHH and within windows enriched for domestic alleles in LA inference are candidate genes for adaptive introgression. P2RY214 was found to be overexpressed in cows with high follicle content, a characteristic that seems to be linked with fertility (Favoreto et al. 2019). The gene was included in the gene network influencing the 305-day milk yield in Guzerá cows, a trait that is very important in dairy cow production and that correlated with reproduction precocity (Paiva et al. 2020). Another study on cow and lactation found several significant SNPs close to this gene to be associated with protein yield in an American cattle breed (Pedrosa et al. 2021). Even in sheep, this gene was found in the list of genes of two enriched GO categories after identifying candidate regions under selection in a breed that has a long history of selection for dairy production (Ruiz-Larrañaga et al. 2018). MED12L is found to be a key regulatory element in pig immunocompetence (Crespo-Piazuelo et al. 2021). In sheep, this gene was found in a region under selection when comparing uniparous and multiparous breeds (Wang et al. 2020); the gene was found in the KEGG Thyroid hormone signaling pathway, that the authors report as important in reproduction. In cows, an intronic variant in MED12L was found to be associated with weaning weight (Londoño-Gil et al. 2021). The evidence from literature suggests that fertility in the introgressed Sardinian WB could be enhanced by the acquisition of domestic alleles at these genes. More experimental proof is needed to assertively confirm this hypothesis, but our analyses are certainly suggestive and call the attention on the possible demographic consequences if domestic regions that confer a reproductive advantage spread in the WB population.

The WB is a very adaptable species, inhabiting five continents (Keuling et al. 2018) and being able to adjust its strategies with the environment (Podgórski et al. 2013; Brogi et al. 2021). The acquisition of advantageous alleles from the DP can increase its plasticity and reproductive success even more. Here, we showed that the level of anthropogenic hybridization in our sample of Sardinian WB is not as high as expected, but the evolutionary trajectory of the population is difficult to predict. We should further expand the sampling areas to identify possible hotspot of hybridization and monitor across time the spread of domestic alleles. Hybridization with the domestic form is common in many species, e.g., wolves, wild horses (Lau et al. 2009; Pilot et al. 2018); humans can have a great impact in the creation of chances for this form of anthropogenic hybridization to happen (Le Roux et al. 2015), especially by releasing hybrids into wild populations (Castillo et al. 2008; Casas et al. 2012), but the retention of domestic alleles and the effects on fitness are hardly foreseeable. Our findings are in line with a study on Eurasian wolf *×* dog admixture that identified a limited number of genomic regions with overrepresented hybrid ancestry to be under selection (Pilot et al. 2021). Even though caution is always recommended, it could be that the overall effect of domestic introgression into wild populations has not the huge impact to genetic integrity, as previously thought. Further genomic investigations should however be extended to other introgressed populations to understand to what extent our results can be generalized.

## Supplementary information

ESM 1

ESM 2

ESM 3
