# Supplementary material for: Anthropogenic hybridization and its influence on the adaptive potential of the Sardinian wild boar (Sus scrofa meridionalis)
Source: J Appl Genet. 2023 Jun 28;64(3):521–30. doi: 10.1007/s13353-023-00763-x (PMC10457222; doi:10.1007/s13353-023-00763-x)
Supplement: Supplementary file 2 — ESM 2 [file 13353_2023_763_MOESM2_ESM.docx]

**Anthropogenic hybridization and its influence on the adaptive potential of the Sardinian wild boar (*Sus scrofa meridionalis*)**

Journal of Applied Genetics

Giulia Fabbri, Ludovica Molinaro, Nadia Mucci, Luca Pagani, and Massimo Scandura

Affiliation corresponding author: Department of Veterinary Medicine, University of Sassari, Via Vienna 2A, 07100 Sassari, Italy

Email corresponding author: g.fabbri@studenti.uniss.it

**Supplementary Text**

***Randomization on the Population Branch Statistics (PBS) analysis***

We performed the same analysis on two sets of 1000 random replicates of pure Sardinian WB having equal size to Hybrid_SarWB. For the first set of replicates we randomly selected the target number of individuals 1000 times leaving the remaining at each run to represent the pure pool; for the second set we simulated the situation of our true introgressed group. This was done to possibly take into account the internal population structure of the Sardinian WB (Fabbri et al. 2022; Lecis et al. 2022). We averaged the number of significant windows obtained with the randomization and compared the windows obtained in our true analysis against the significant ones in the randomization sets.

***XP-EHH analysis in rehh R package***

We set ‘maxgap = 200,000’ to account for the possible bias introduced by large gaps in the medium density SNPchip. We computed the one-sided p-value because we were interested in the regions under selection in pop1 (DP) and not in pop2 (SarWB). We further identified candidate windows under selection in DP but not in the unadmixed Sardinian WB with the function ‘calc_candidate_regions’ if at least two SNPs were above the threshold (that we set at XP-EHH = 3) with a window size of 500 kbp, a step size of 250 kbp, and the argument ‘join_neighbors = T’.

***Haplotype structure analysis***

As one region resulted significant in the two selection analyses but was not enriched for domestic alleles according to ELAI, we applied a method to analyze the haplotype structure of the whole dataset around this region. In particular, Haplostrips (Marnetto and Huerta-Sánchez 2017) needs a phased dataset and outputs a matrix where haplotypes are ordered according to their similarity, such that the two most divergent haplotypes are found at the first and last rows. We checked the location of the haplotypes from the Hybrid_SarWB samples with respect to SarWB and the various DP breeds.

**References**

Fabbri G, Iacolina L, Apollonio M, Scandura M (2022) The Role of the Environment in Shaping the Genomic Variation in an Insular Wild Boar Population. Diversity 14:1–17. https://doi.org/10.3390/d14090774

Lecis R, Dondina O, Orioli V, et al (2022) Main roads and land cover shaped the genetic structure of a Mediterranean island wild boar population. Ecol Evol 12:e8804. https://doi.org/10.1002/ECE3.8804

Marnetto D, Huerta-Sánchez E (2017) Haplostrips: revealing population structure through haplotype visualization. Methods Ecol Evol 8:1389–1392. https://doi.org/10.1111/2041-210X.12747


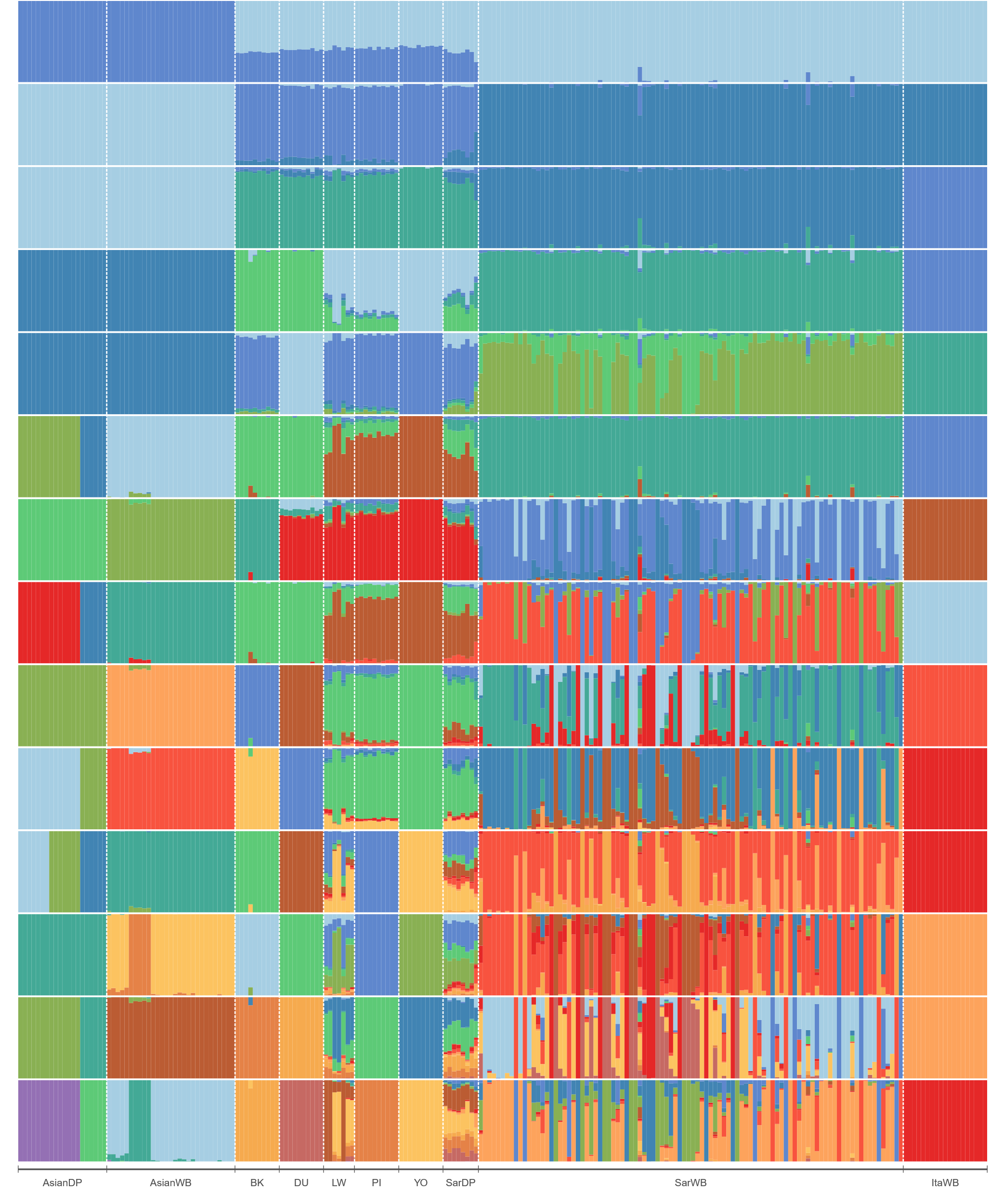


**Fig. S1** ADMIXTURE plot for the whole dataset analyzed in this study. Description: The number of ancestral populations tested went from K = 2 to K = 15. SarWB = Sardinian wild boar (*Sus scrofa meridionalis*); ItaWB = Italian WB; AsianWB = Asian WB; BK = Berkshire pig breed; DU = Duroc pig breed; LW = Large White pig breed; PI = Pietrain pig breed; YO = Yorkshire pig breed; SardDP = Sardinian local pig breed AsianDP = Meishan pig breed from Asia


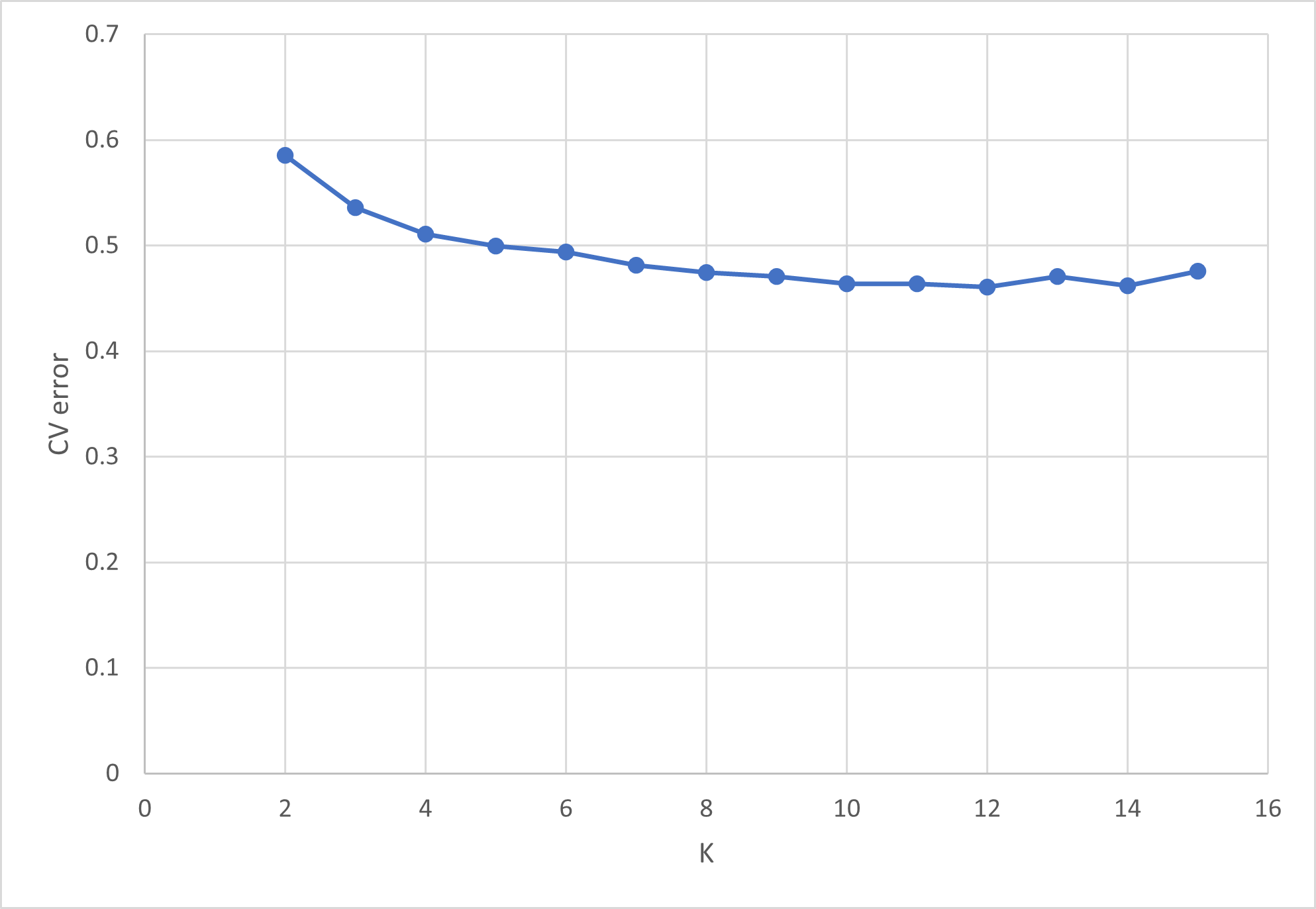


**Fig. S2** Cross-validation error computed with ADMIXTURE for K = 2 to K = 11.

**
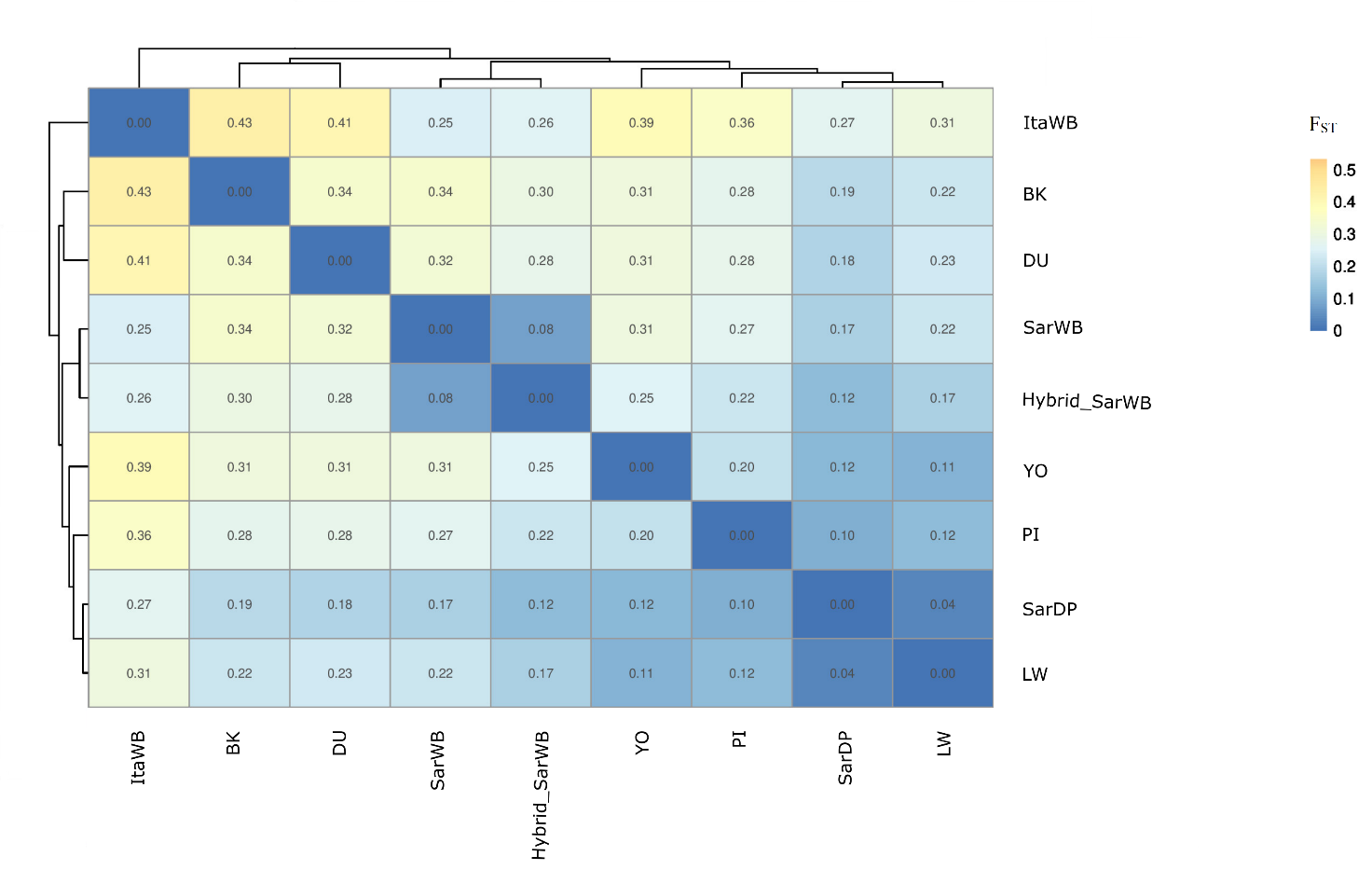
Fig. S3** Pairwise F_ST_ values. The color code of the heatmap reports the higher F_ST_ values in red and the lower in blue. A dendrogram at the margin of the matrix defines the relationships among the clusters. SarWB = Sardinian wild boar (*Sus scrofa meridionalis*); Hybrid_SawWB = Sardinian wild boar with clear signs of hybridization with the domestic pig; BK = Berkshire pig breed; DU = Duroc pig breed; LW = Large White pig breed; PI = Pietrain pig breed; YO = Yorkshire pig breed; SardDP = Sardinian local pig breed. The least divergent F_ST_ values were found when comparing SarWB-Hybrid_SarWB and SarDP-LW. Following the PCA and ADMIXTURE results, we separated for the F_ST_ calculation the outliers from their cluster, defining two SarWB groups (SarWB and Hybrid_SarWB).


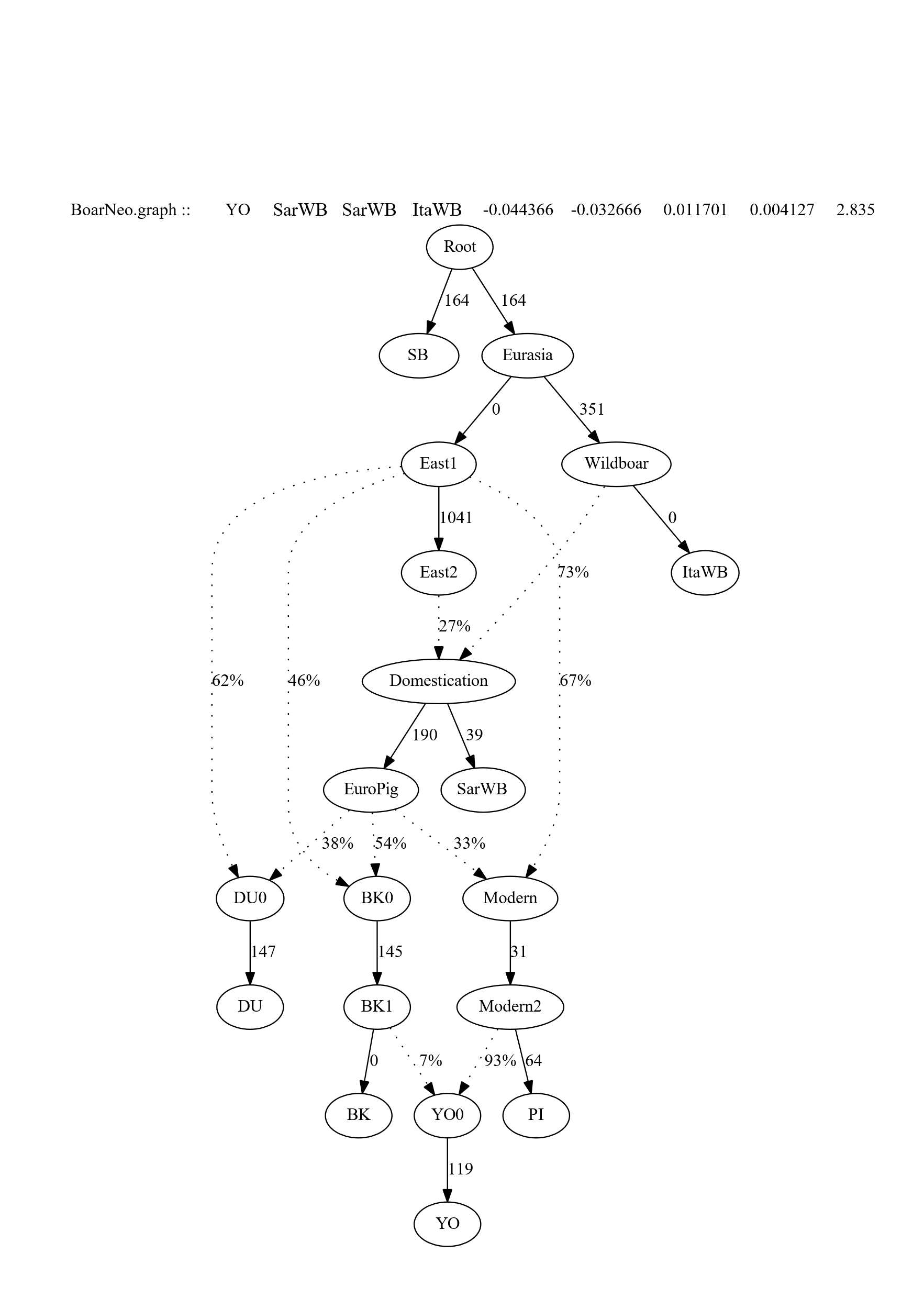


**Fig. S4** Admixture graph on the studied populations. The graph modeled the position of the Sardinian wild boar population in relation with another wild boar population and a range of domestic pig breeds. The model is sustained by a Z-score < |3|.

**
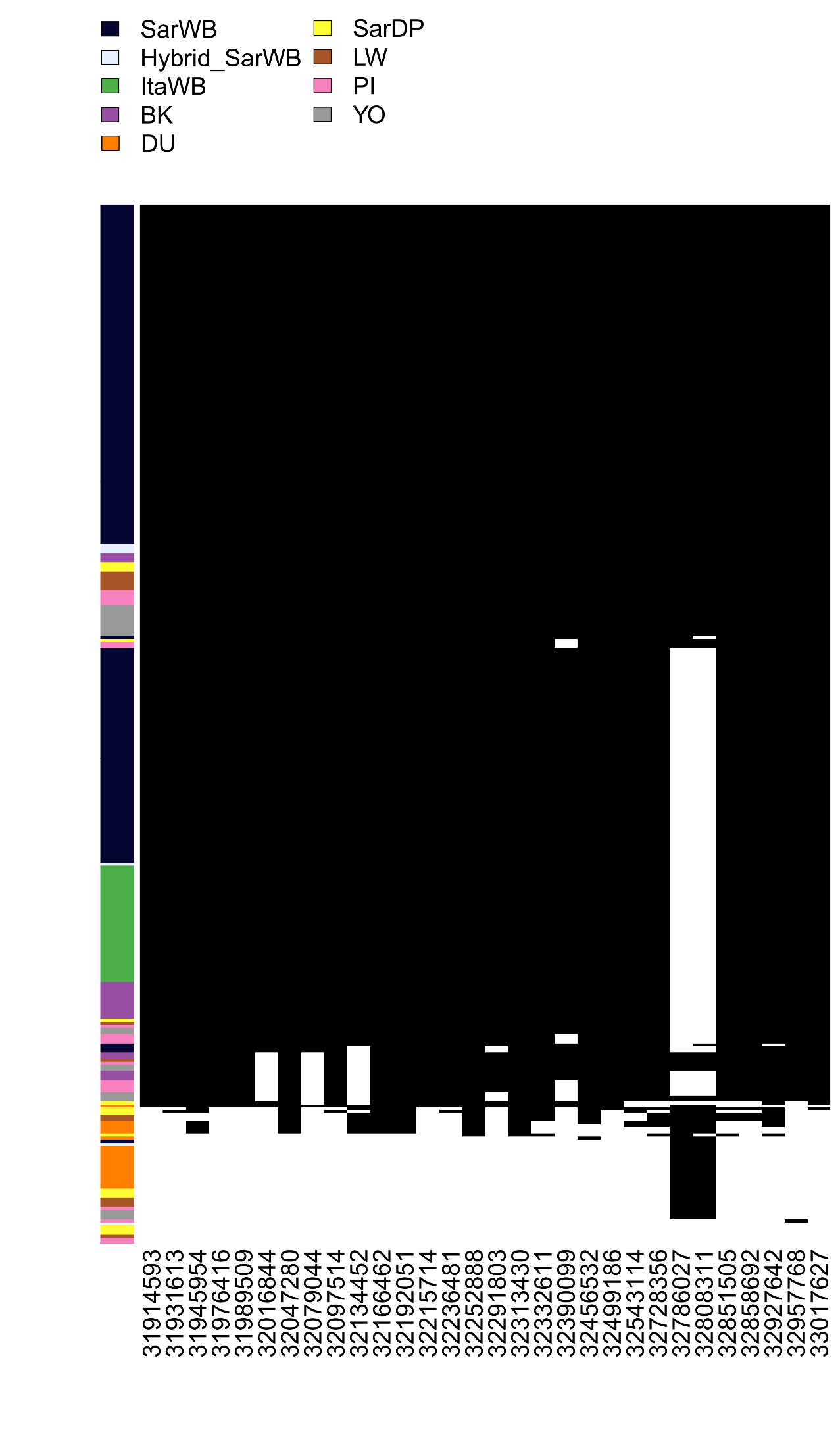
**

**Fig. S6** Haplostrips plot of the region spanning 31.9 to 33.0 Mbp on chromosome 7. The region resulted as significant in both the population branch statistics analysis and in the XP-EHH analysis. Four out of six haplotypes making up the hybrid Sardinian wild boar group are positioned with the two main haplotypes in the wild populations (i.e. SarWB and ItaWB), while the remaining two clustered within haplotypes more common in the domestic pig breeds. SarWB = Sardinian wild boar (*Sus scrofa meridionalis*); Hybrid_SawWB = Sardinian wild boar with clear signs of hybridization with the domestic pig; BK = Berkshire pig breed; DU = Duroc pig breed; LW = Large White pig breed; PI = Pietrain pig breed; YO = Yorkshire pig breed; SardDP = Sardinian local pig breed; SB = Bornean bearded pig (*S. barbatus*); Outlier_SB = Bornean bearded pig resulted as an outlier in the PCA and with a pig-like component in the ADMIXTURE analysis.

**
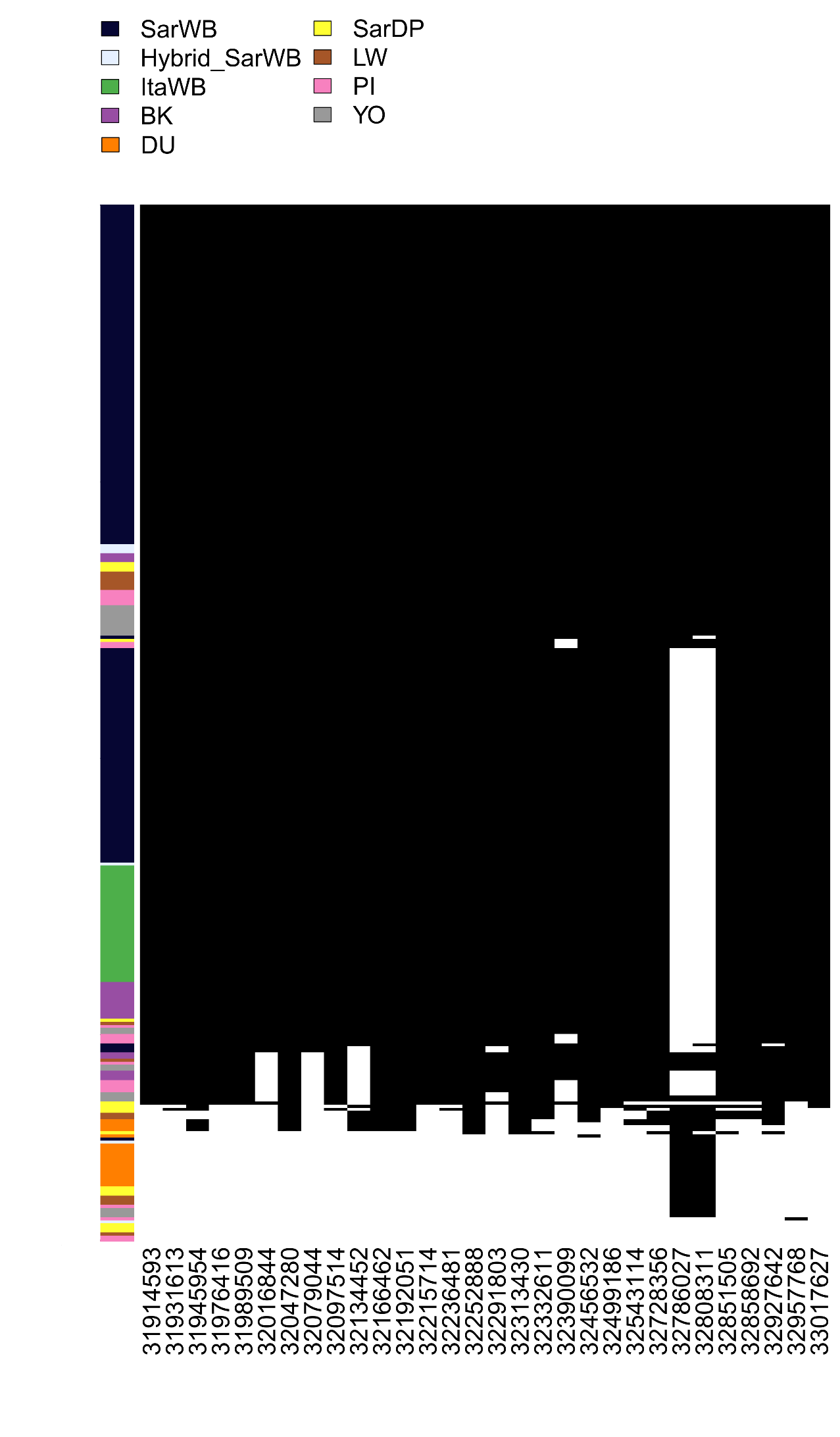
**

**Fig. S7** Haplostrips plot of the region spanning 29.9 to 35.0 Mbp on chromosome 7. We extended the region analyzed in Figure S6 of 2Mbp upstream and downstream to better characterize the composition of the haplotypes in the hybrid Sardinian wild boar in relation to the other wild and domestic groups. Two haplotypes clearly clustered within haplotypes found mainly in domestic pig breeds. SarWB = Sardinian wild boar (*Sus scrofa meridionalis*); Hybrid_SawWB = Sardinian wild boar with clear signs of hybridization with the domestic pig; BK = Berkshire pig breed; DU = Duroc pig breed; LW = Large White pig breed; PI = Pietrain pig breed; YO = Yorkshire pig breed; SardDP = Sardinian local pig breed; SB = Bornean bearded pig (*S. barbatus*); Outlier_SB = Bornean bearded pig resulted as an outlier in the PCA and with a pig-like component in the ADMIXTURE analysis.

**Figure captions in file Online resource 3**

**Fig. S5** Chromosome-wide assignment of each SNP marker to the Boar or Pig ancestry. The ancestry assignment is averaged across the three hybrid Sardinian wild boars. The unlabeled loci did not pass the selected cutoff of 0.9.
