# Supplementary material for: Anthropogenic hybridization and its influence on the adaptive potential of the Sardinian wild boar (Sus scrofa meridionalis)
Source: J Appl Genet. 2023 Jun 28;64(3):521–30. doi: 10.1007/s13353-023-00763-x (PMC10457222; doi:10.1007/s13353-023-00763-x)
Supplement: Supplementary file 3 — ESM 3 [file 13353_2023_763_MOESM3_ESM.pdf]

Chr 1

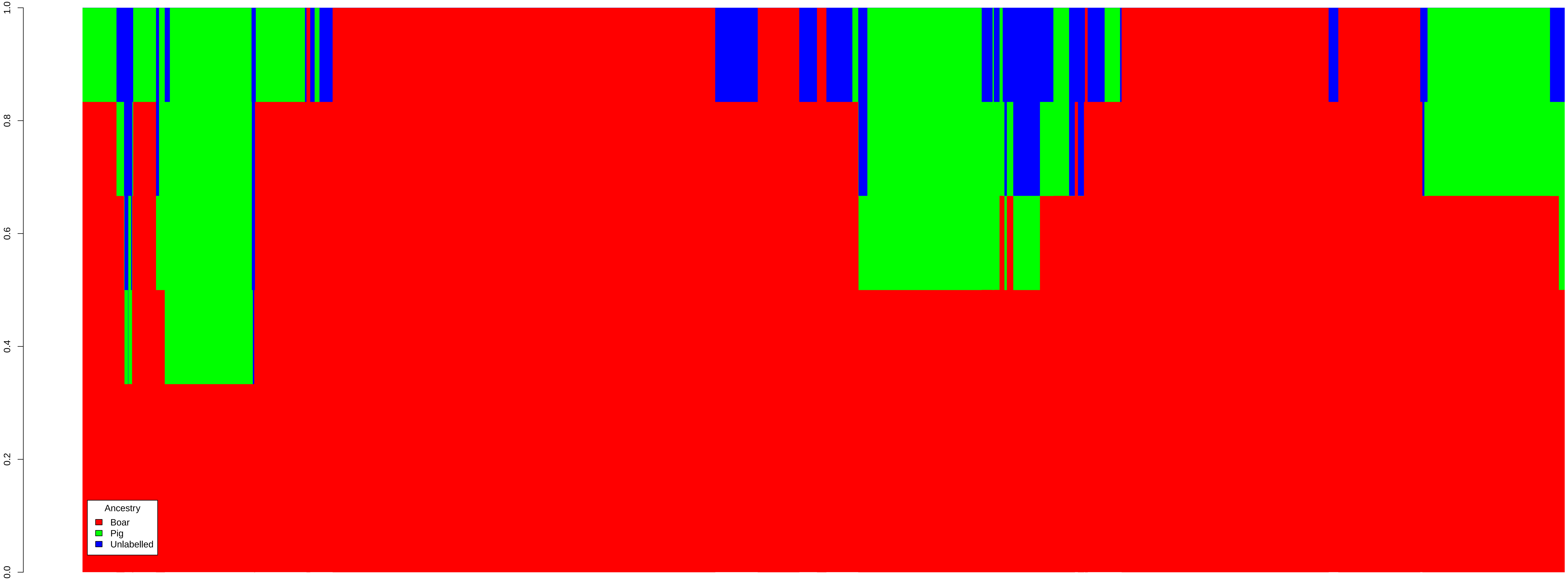

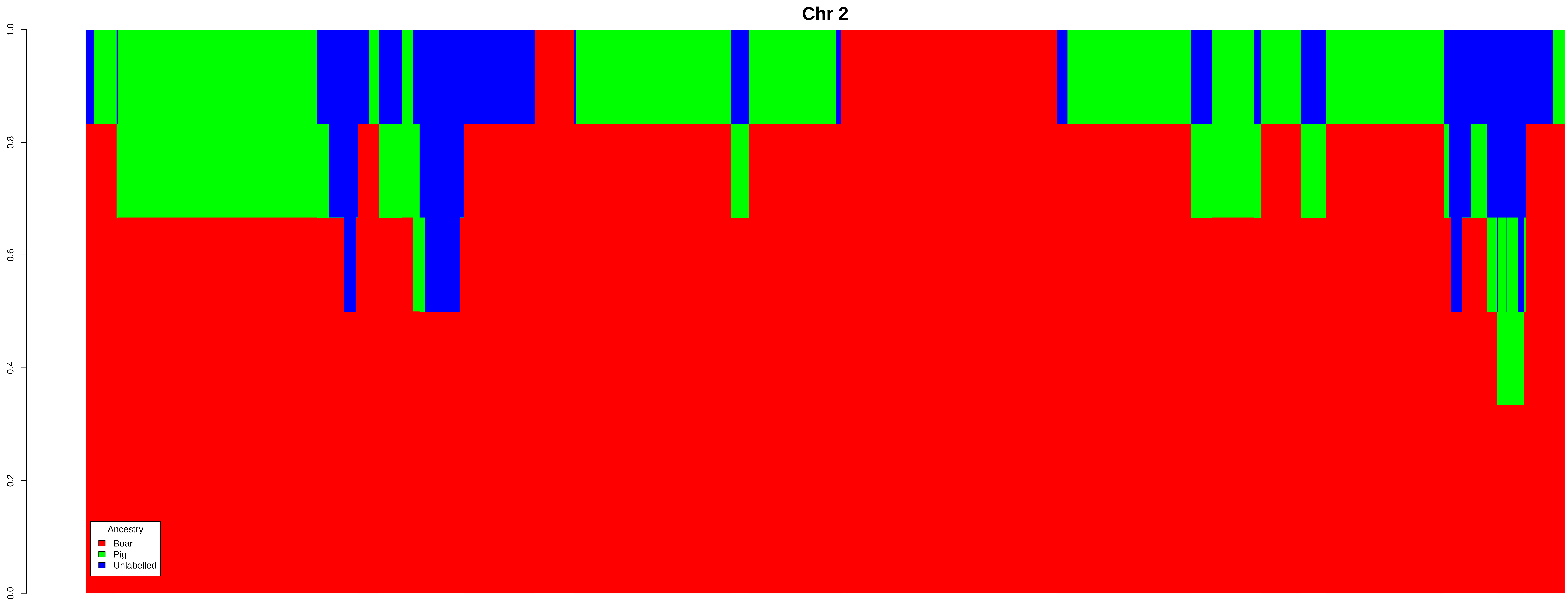
Chr 2
1.0
0.8
0.6
0.4
0.2
0.0
Ancestry
Boar
Pig
Unlabelled

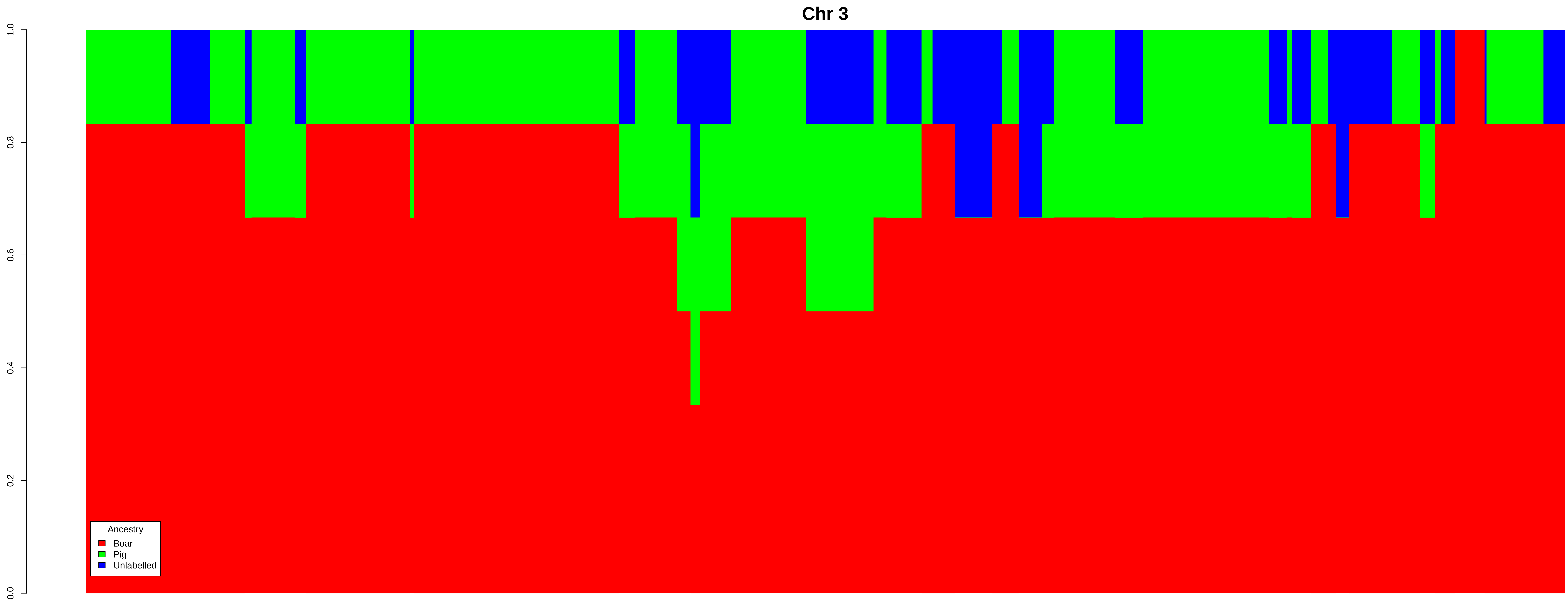

Chr 3
1.0
0.8
0.6
0.4
0.2
0.0
Ancestry
Boar
Pig
Unlabelled

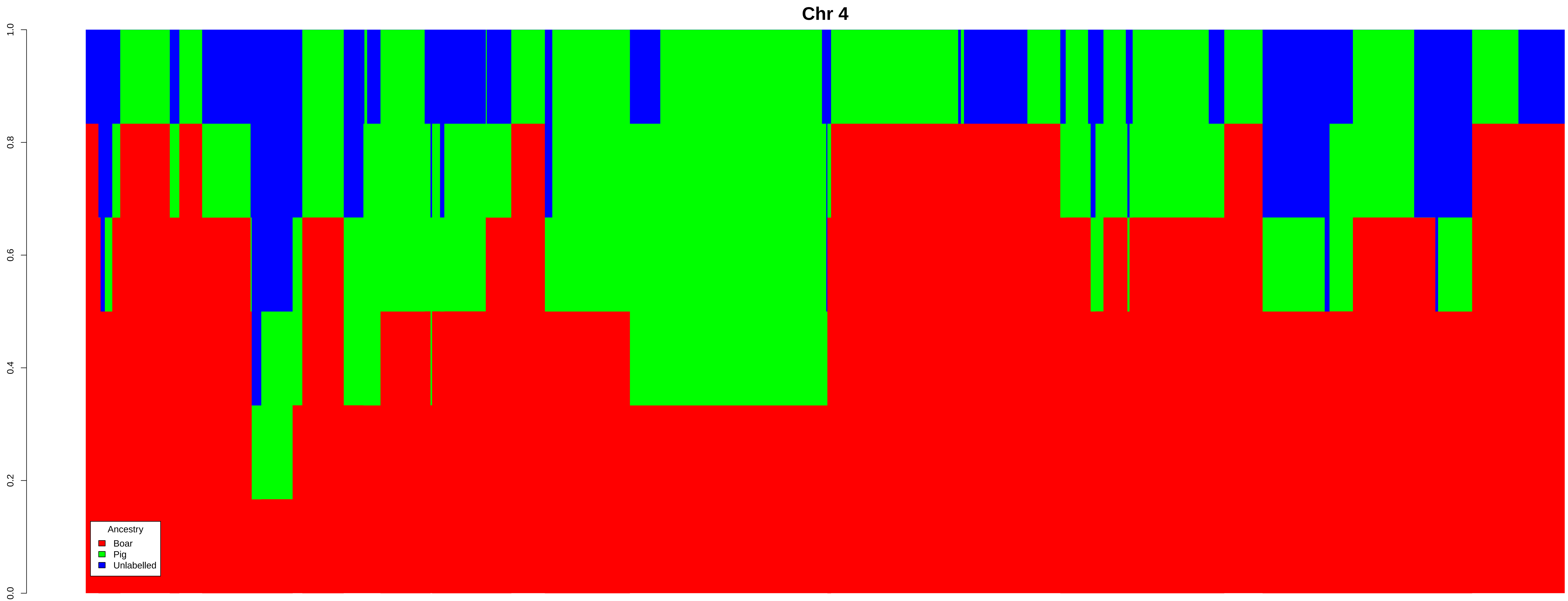

Chr 4
1.0
0.8
0.6
0.4
0.2
0.0
Ancestry
Boar
Pig
Unlabelled

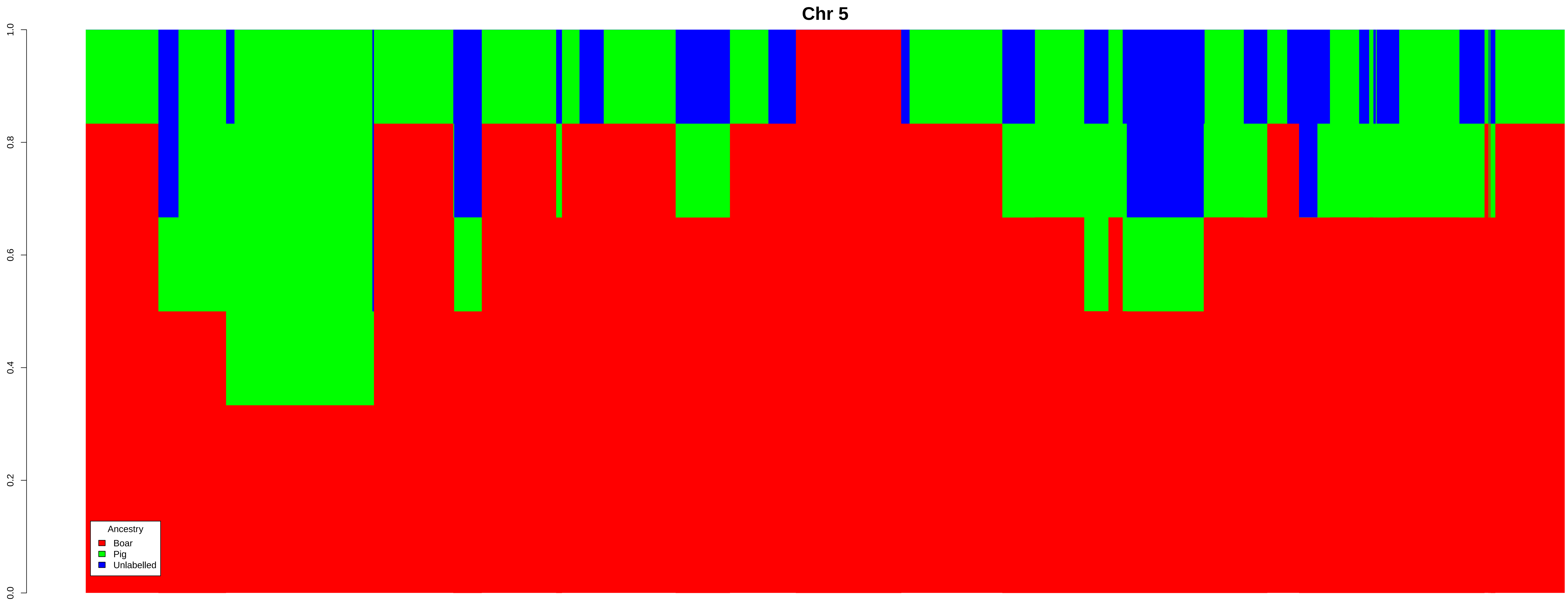

Chr 5
0.0
0.2
0.4
0.6
0.8
1.0
Ancestry
Boar
Pig
Unlabelled

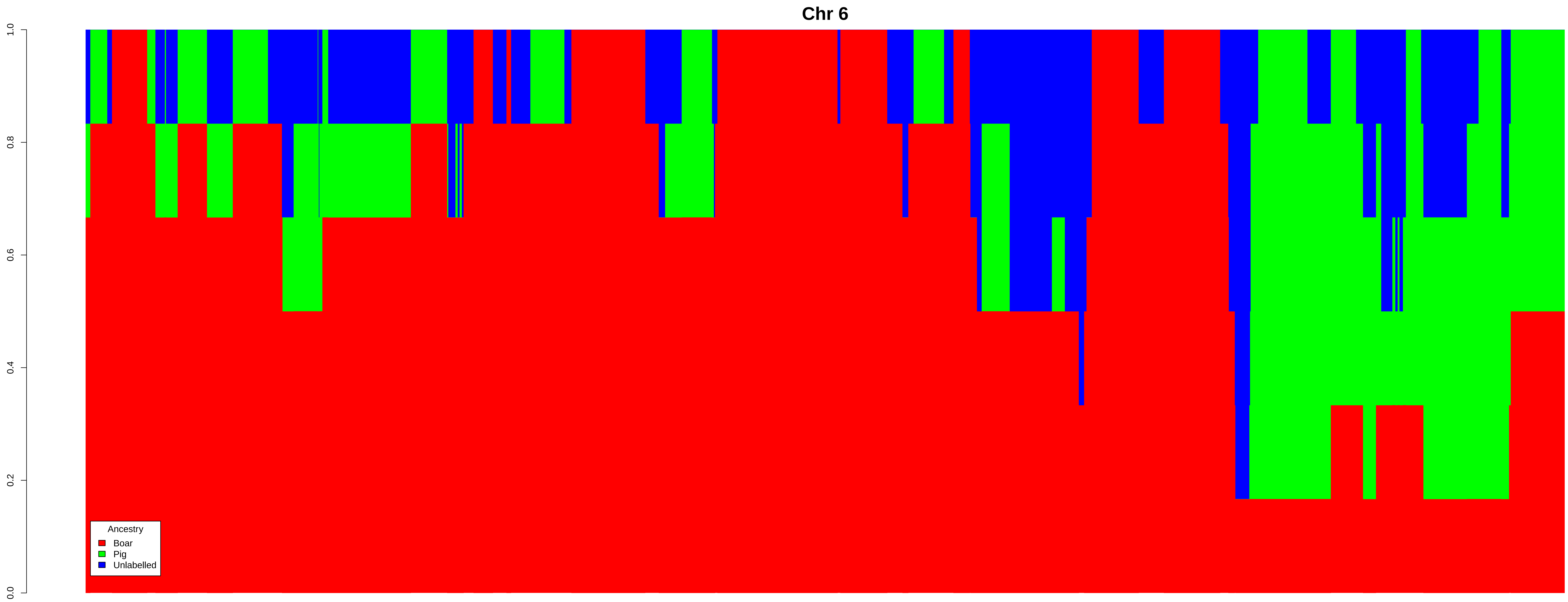

Chr 6
1.0
0.8
0.6
0.4
0.2
0.0
Ancestry
Boar
Pig
Unlabelled

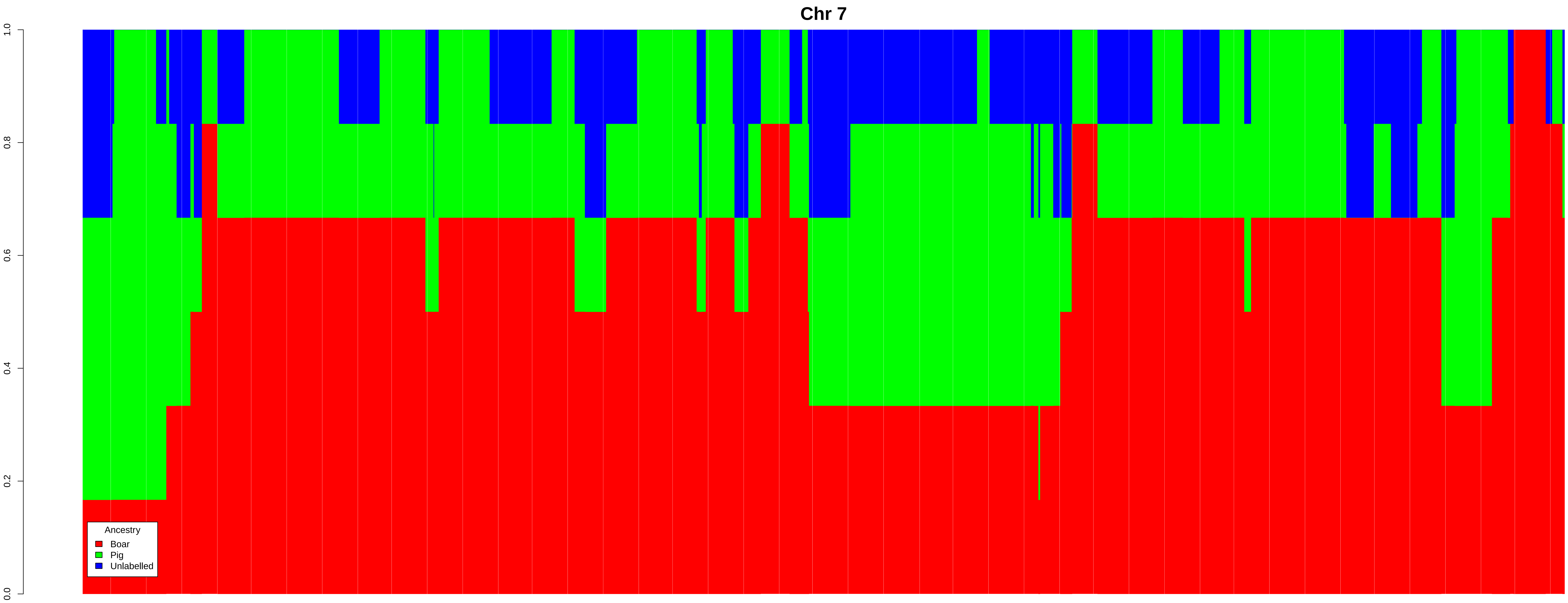
Chr 7
0.0
0.2
0.4
0.6
0.8
1.0
Ancestry
Boar
Pig
Unlabelled

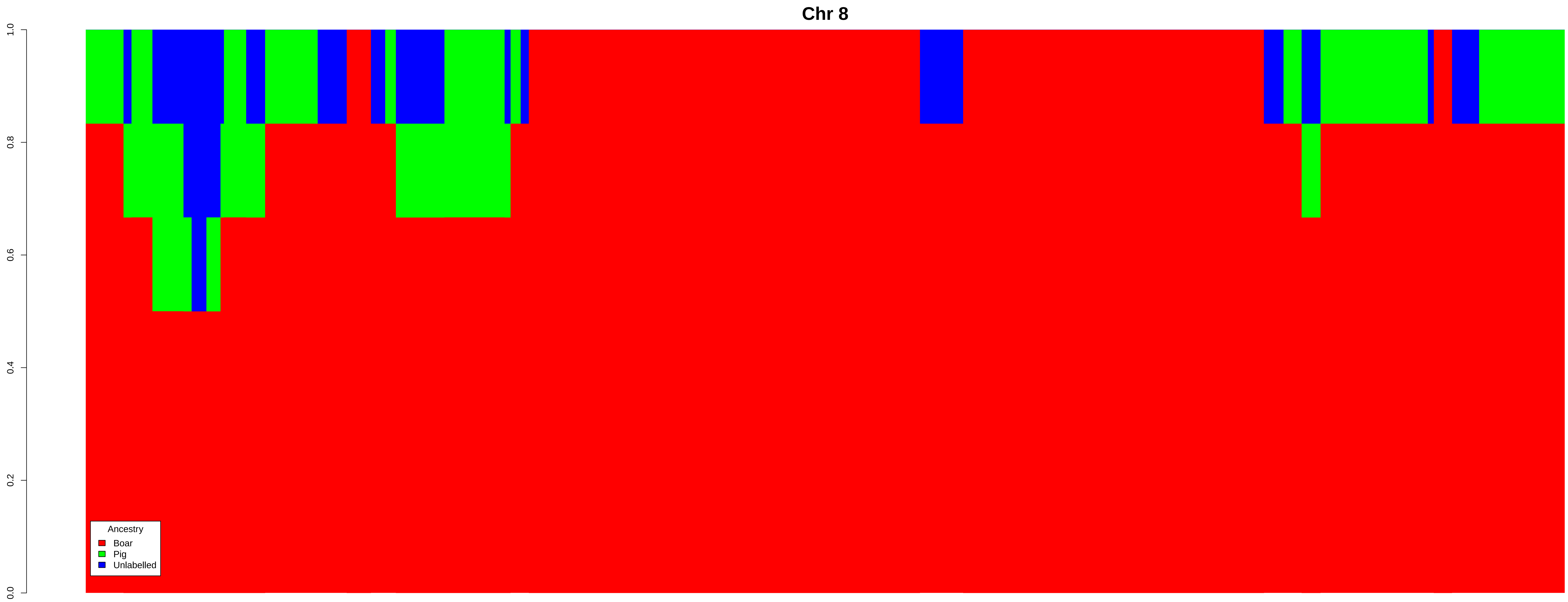
Chr 8
1.0
0.8
0.6
0.4
0.2
0.0
Ancestry
Boar
Pig
Unlabelled

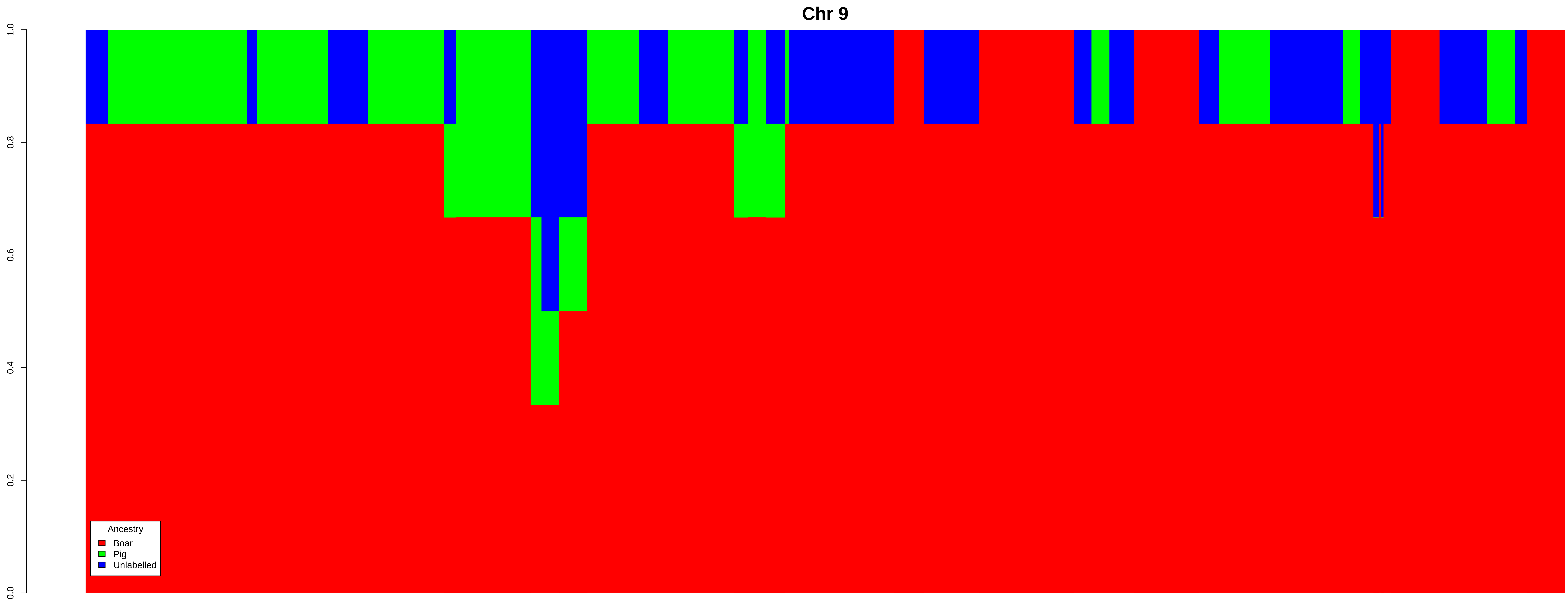

Chr 9
1.0
0.8
0.6
0.4
0.2
0.0
Ancestry
Boar
Pig
Unlabelled

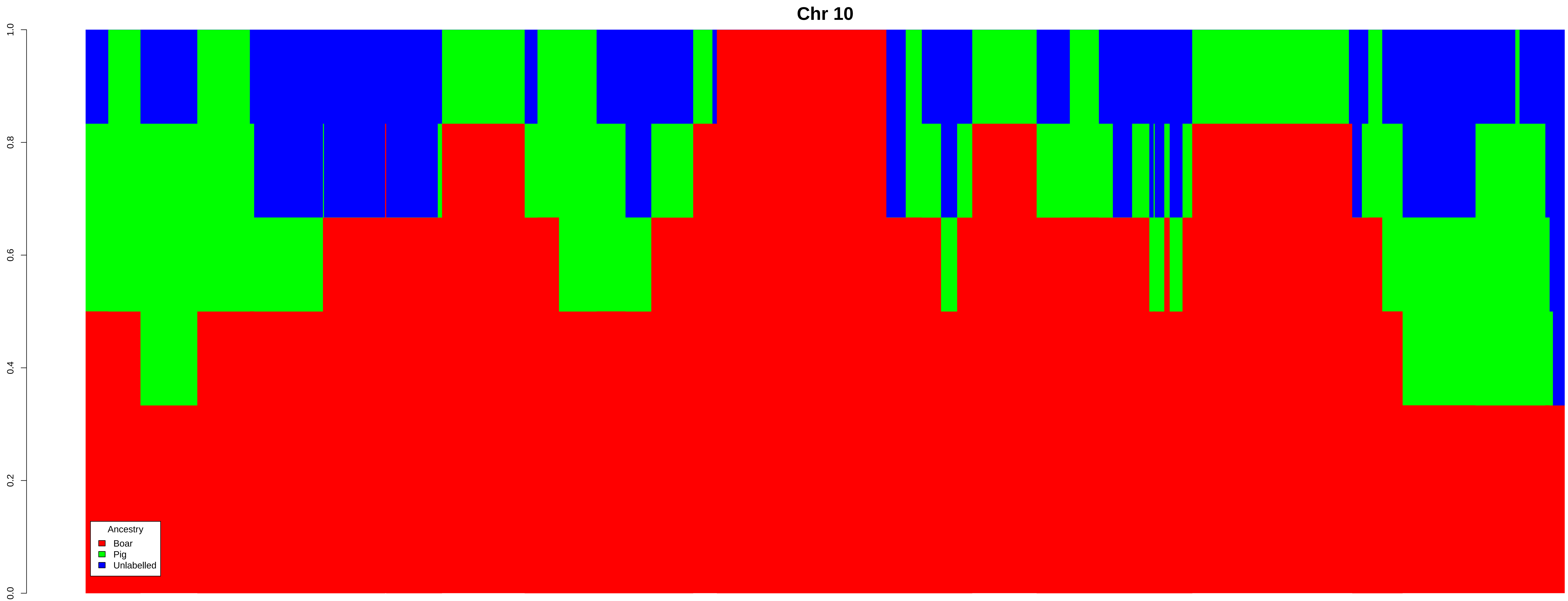
Chr 10
1.0
0.8
0.6
0.4
0.2
0.0
Ancestry
Boar
Pig
Unlabelled

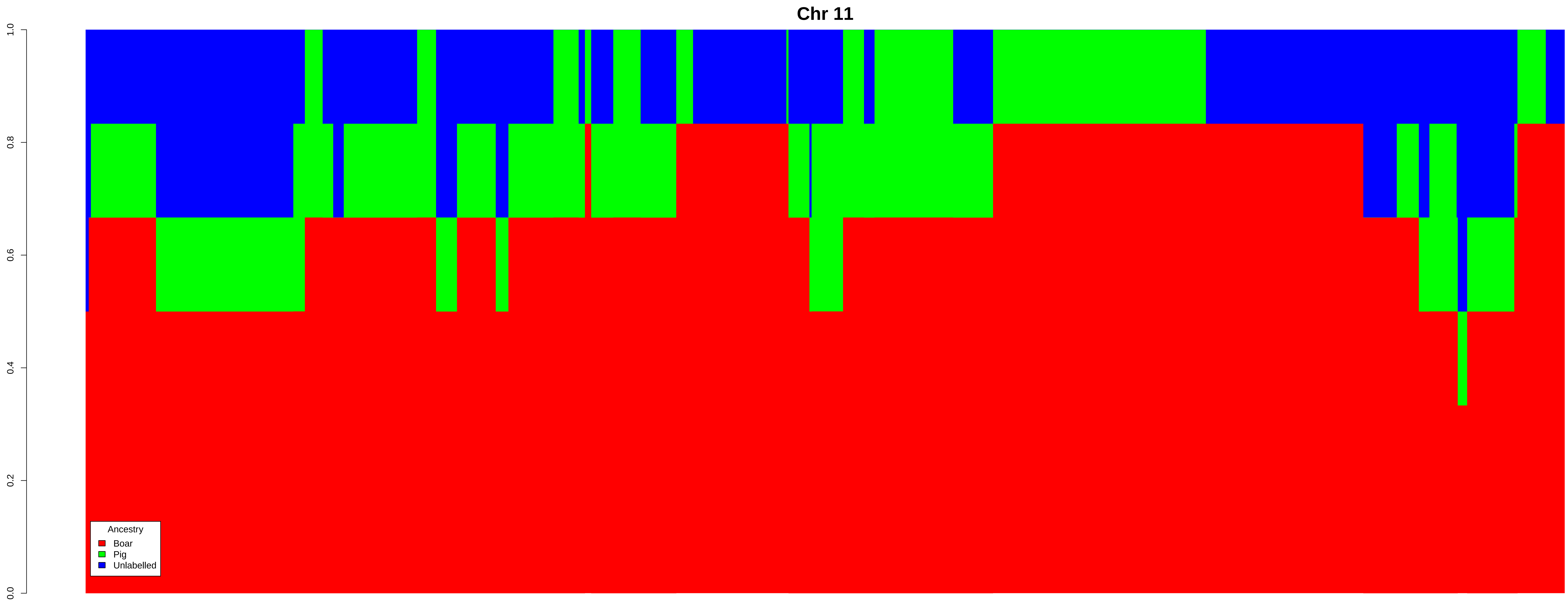
Chr 11
1.0
0.8
0.6
0.4
0.2
0.0
Ancestry
Boar
Pig
Unlabelled

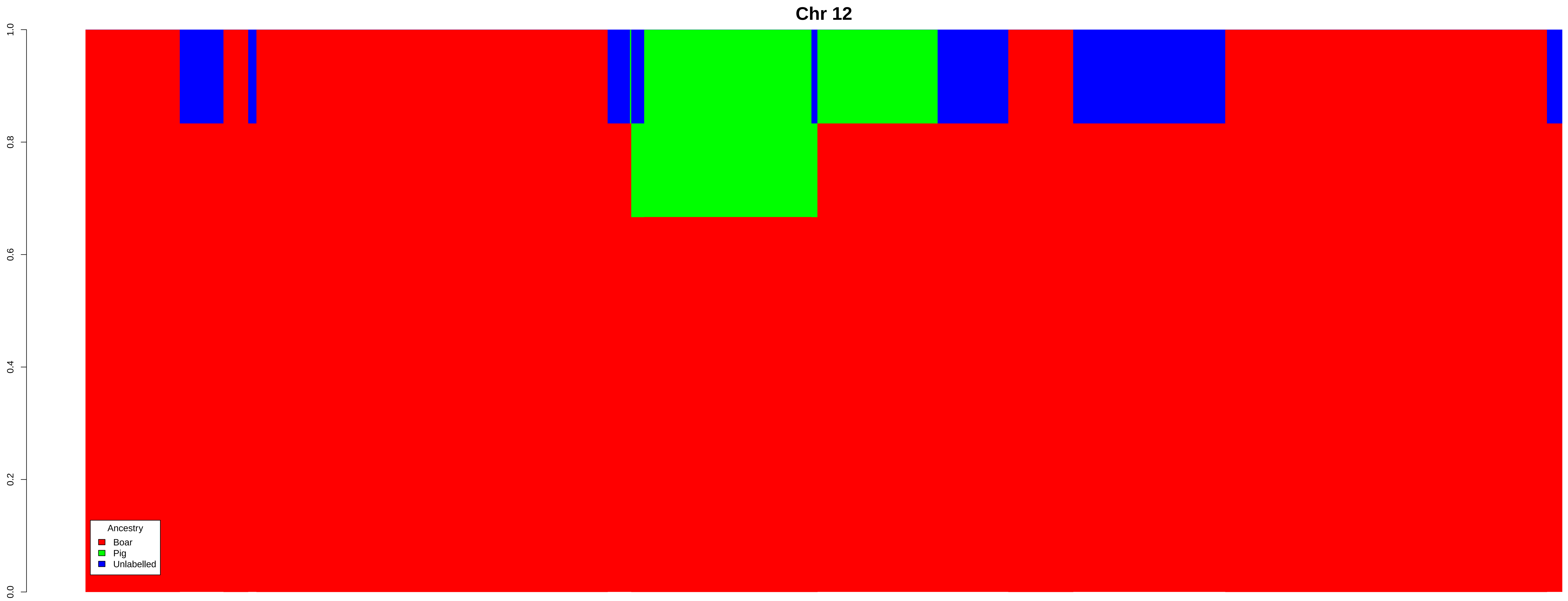
Chr 12
0.0
0.2
0.4
0.6
0.8
1.0
Ancestry
Boar
Pig
Unlabelled

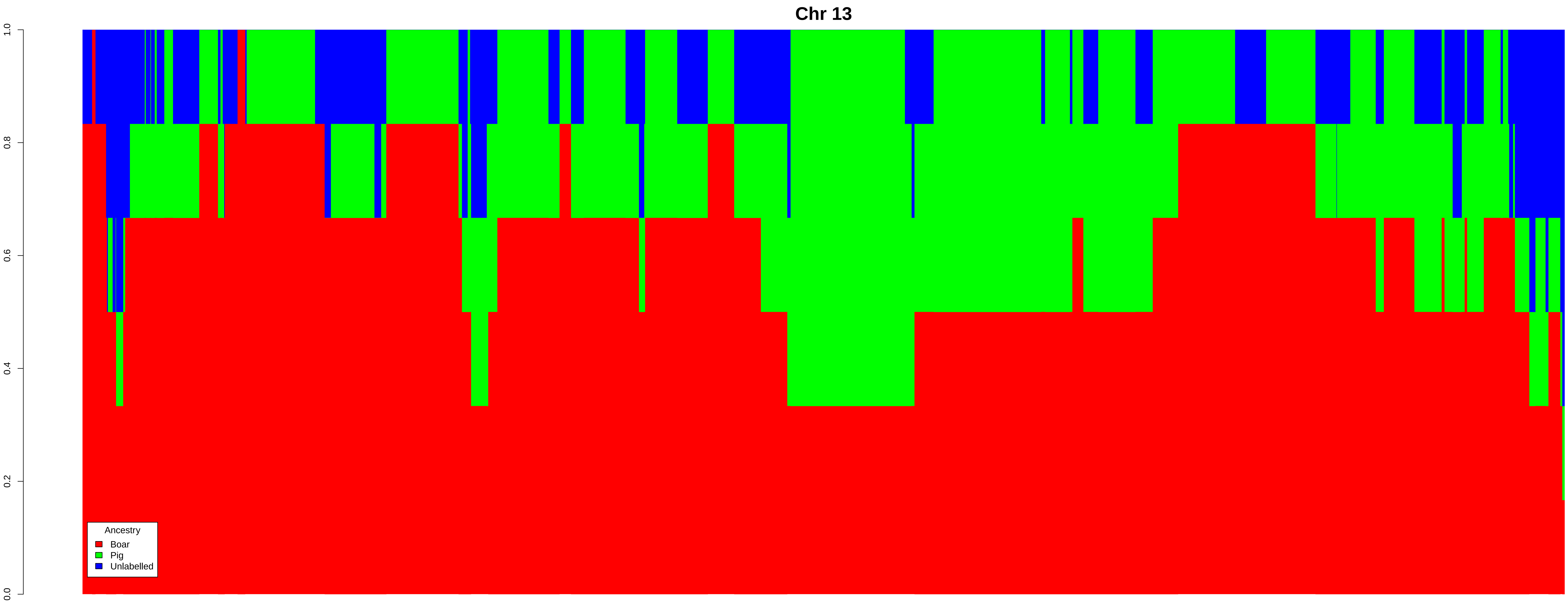
Chr 13
1.0
0.8
0.6
0.4
0.2
0.0
Ancestry
Boar
Pig
Unlabelled

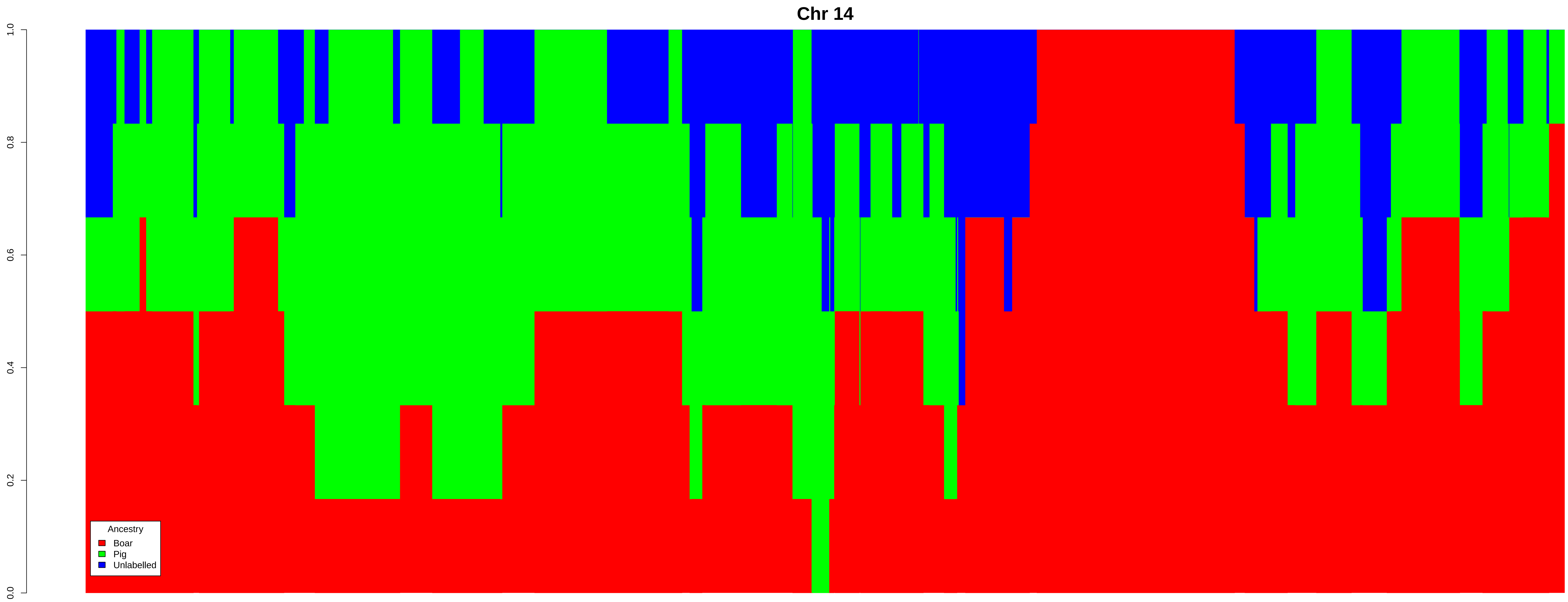

Chr 14
1.0
0.8
0.6
0.4
0.2
0.0
Ancestry
Boar
Pig
Unlabelled

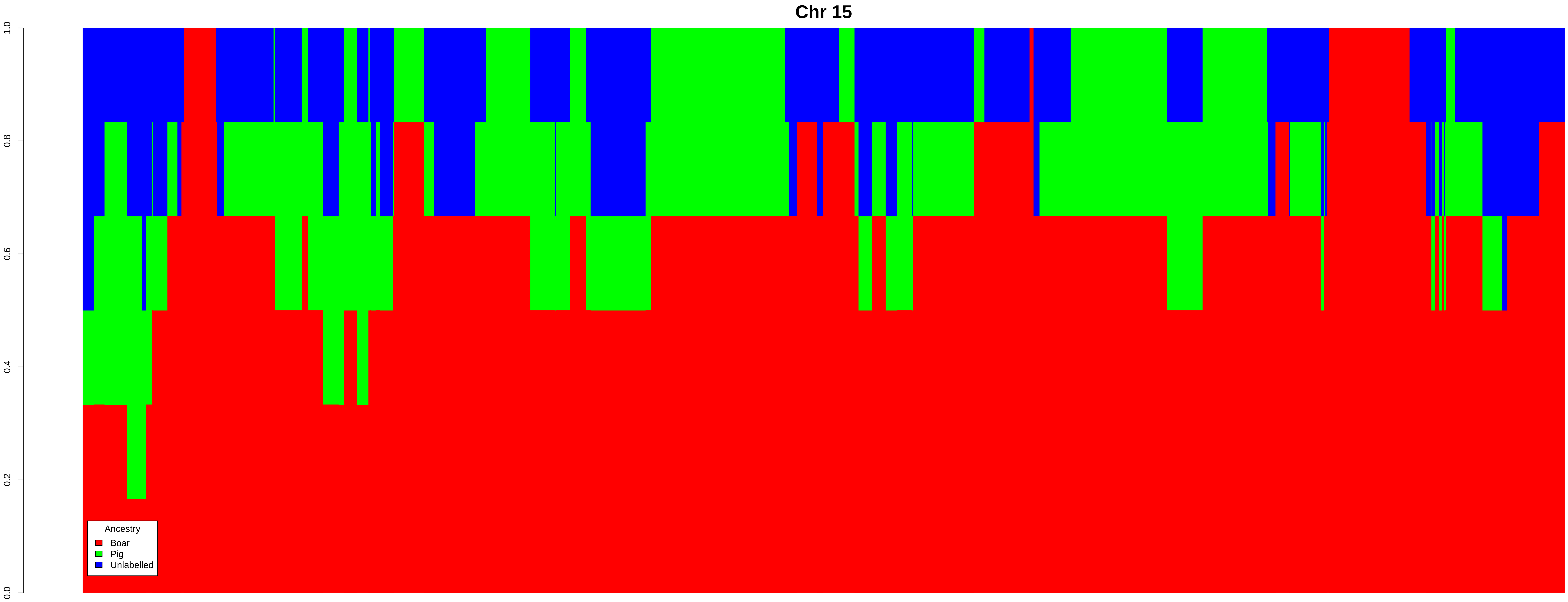

Chr 15
1.0
0.8
0.6
0.4
0.2
0.0
Ancestry
Boar
Pig
Unlabelled

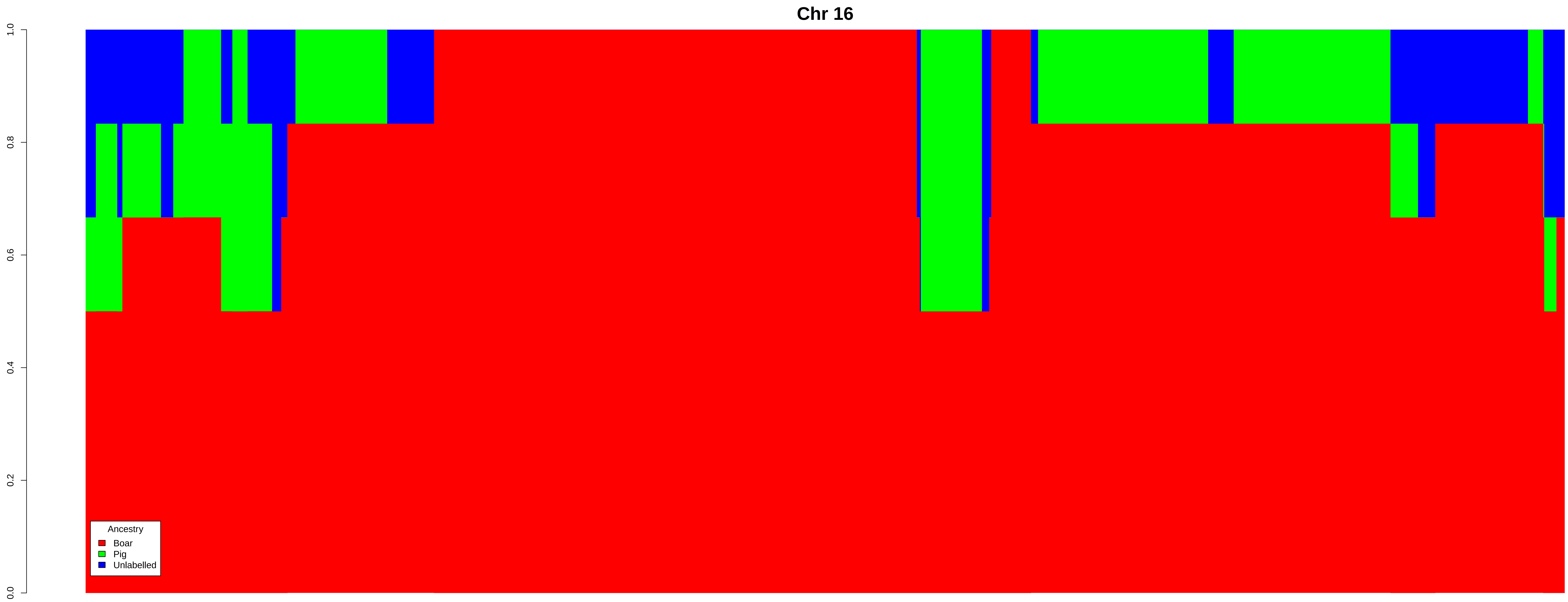
Chr 16
1.0
0.8
0.6
0.4
0.2
0.0
Ancestry
Boar
Pig
Unlabelled

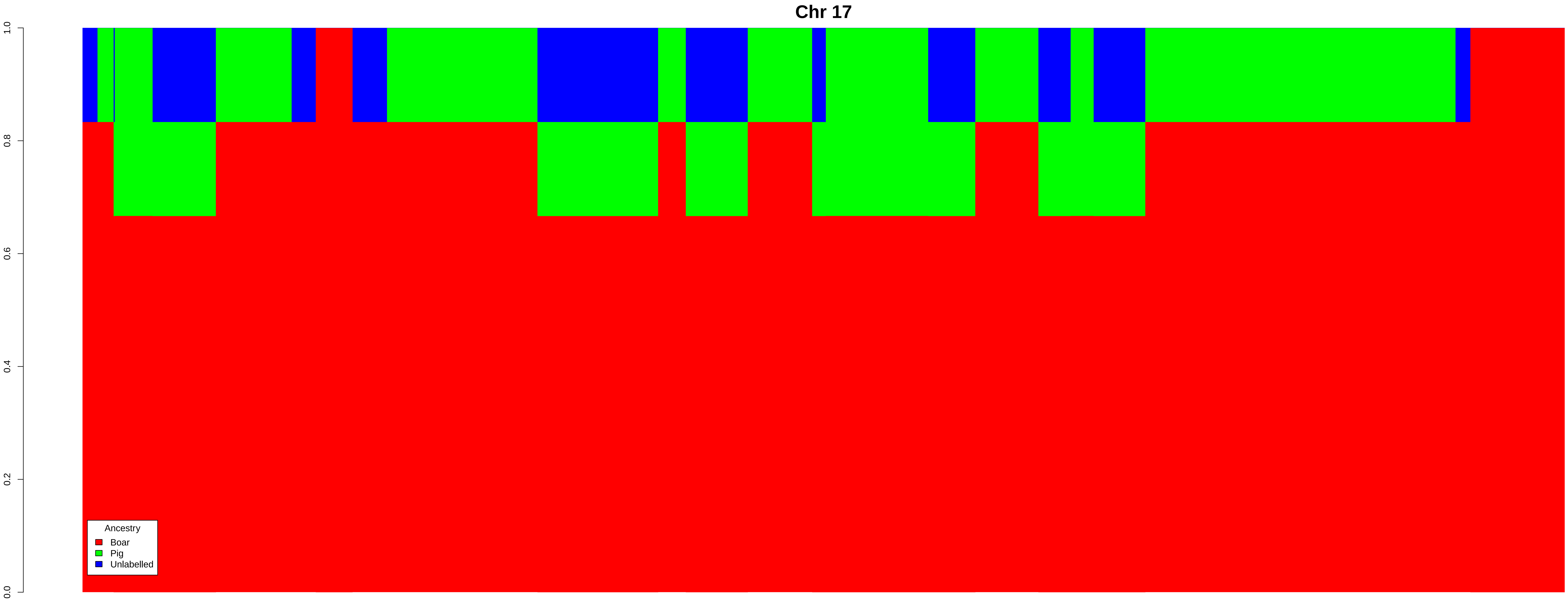

Chr 17
1.0
0.8
0.6
0.4
0.2
0.0
Ancestry
Boar
Pig
Unlabelled

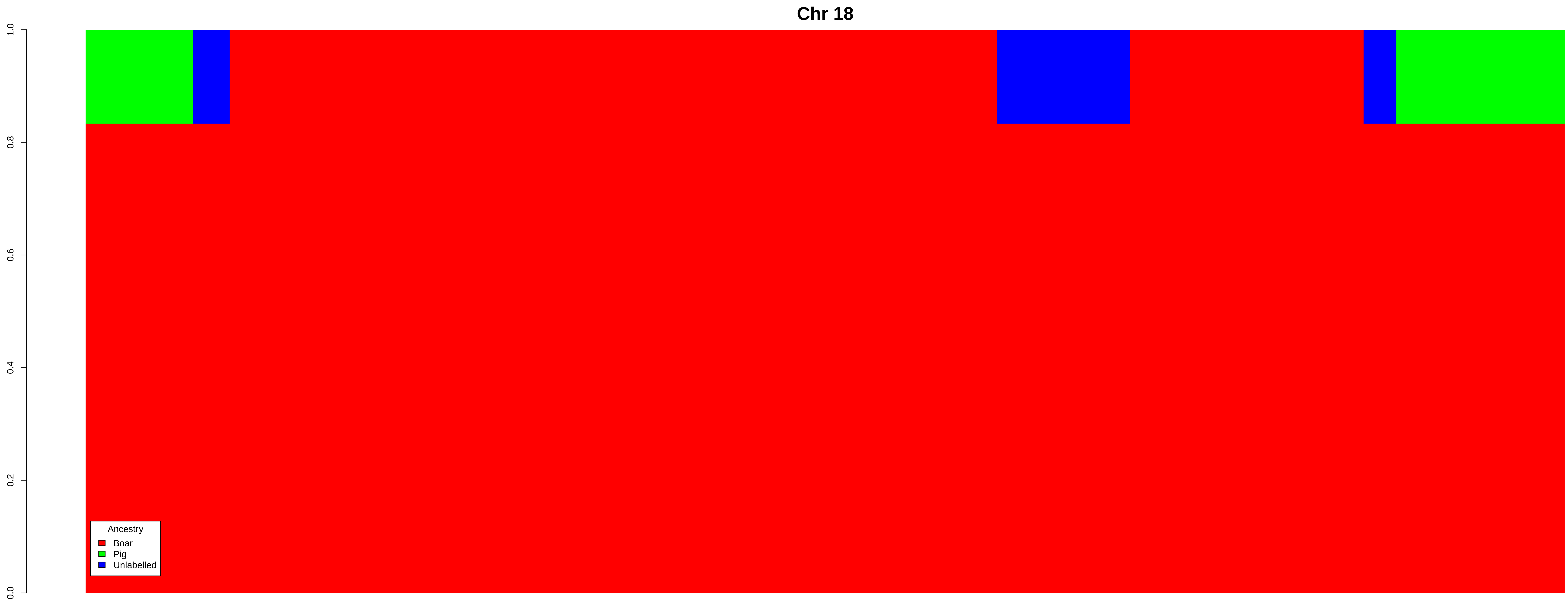
Chr 18
1.0
0.8
0.6
0.4
0.2
0.0
Ancestry
Boar
Pig
Unlabelled
